# Registration of spatio-temporal point clouds of plants for phenotyping

**DOI:** 10.1371/journal.pone.0247243

**Published:** 2021-02-25

**Authors:** Nived Chebrolu, Federico Magistri, Thomas Läbe, Cyrill Stachniss

**Affiliations:** Photogrammetry and Robotics Lab, University of Bonn, Bonn, Germany; Institut de Robotica i Informatica Industrial, SPAIN

## Abstract

Plant phenotyping is a central task in crop science and plant breeding. It involves measuring plant traits to describe the anatomy and physiology of plants and is used for deriving traits and evaluating plant performance. Traditional methods for phenotyping are often time-consuming operations involving substantial manual labor. The availability of 3D sensor data of plants obtained from laser scanners or modern depth cameras offers the potential to automate several of these phenotyping tasks. This automation can scale up the phenotyping measurements and evaluations that have to be performed to a larger number of plant samples and at a finer spatial and temporal resolution. In this paper, we investigate the problem of registering 3D point clouds of the plants over time and space. This means that we determine correspondences between point clouds of plants taken at different points in time and register them using a new, non-rigid registration approach. This approach has the potential to form the backbone for phenotyping applications aimed at tracking the traits of plants over time. The registration task involves finding data associations between measurements taken at different times while the plants grow and change their appearance, allowing 3D models taken at different points in time to be compared with each other. Registering plants over time is challenging due to its anisotropic growth, changing topology, and non-rigid motion in between the time of the measurements. Thus, we propose a novel approach that first extracts a compact representation of the plant in the form of a skeleton that encodes both topology and semantic information, and then use this skeletal structure to determine correspondences over time and drive the registration process. Through this approach, we can tackle the data association problem for the time-series point cloud data of plants effectively. We tested our approach on different datasets acquired over time and successfully registered the 3D plant point clouds recorded with a laser scanner. We demonstrate that our method allows for developing systems for automated temporal plant-trait analysis by tracking plant traits at an organ level.

## Introduction

For optimizing any process, it is important to know or observe the current status of the system to optimize. Thus, approaches for observing or monitoring dynamic systems over extended periods of time are of key interest in several disciplines, especially when dealing with complex systems where input-output relations are complex to formalize. In plant sciences and modern agriculture high-resolution monitoring of plants plays an important role [1, 2]. It forms the basis for analyzing crop performance and provides an indicator of the plant stresses. Measuring how individual plants develop and grow over time is often a manual and laborious task. It often even requires invasive methods that harm the crop. For example, the standard approach to measuring the leaf area is to cut off the leaves and scan them with a flatbed scanner. New measurement technologies for measuring and tracking phenotypic traits employing robots and robotic sensors open up new possibilities to automate the process of measuring plant performance [3, 4]. Recent studies [5, 6] showcase the potential of using 3D laser data for computing geometric plant traits with high fidelity. Agricultural robots equipped with such laser scanners can acquire 3D plant data at a large scale and facilitate high-resolution phenotyping. This is a step forward for scaling up phenotyping from plants grown in a greenhouse to the level of plots and maybe even fields.

**Fig 1:**
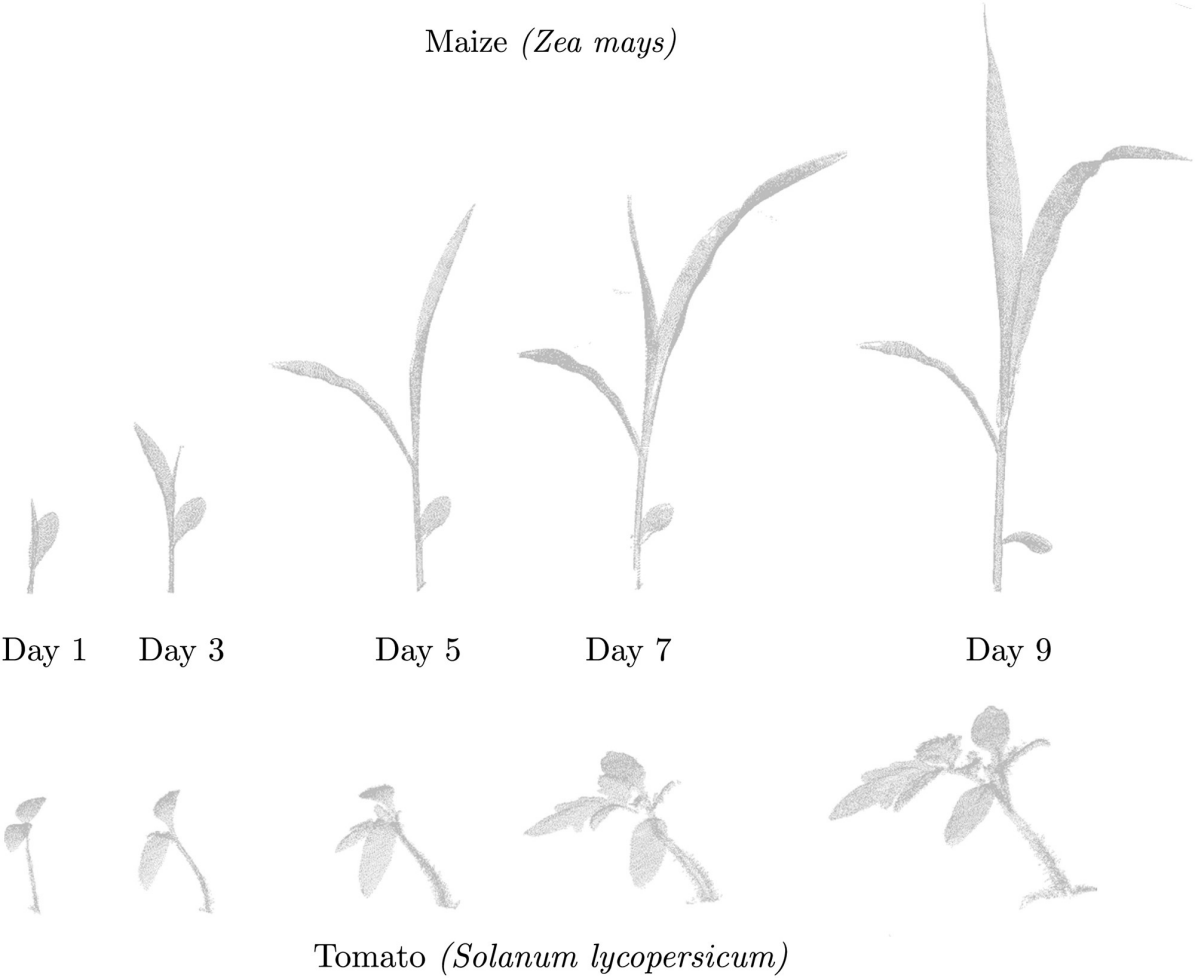
A time-series of 3D point clouds of two plants (maize (top) and tomato (bottom)) captured during its growth. Our goal is to develop techniques for automatically registering such 3D scans captured under challenging conditions of changing topology and anisotropic growth of the plant.

Laser scanners and RGB-D cameras, i.e., sensors that can measure the 3D geometry of objects, are commonly used sensors to perceive both static and dynamic environments. Especially in robotics, this is a key task that most mobile systems rely on [7–9]. Extending such robotic-inspired approaches to the agricultural setting, however, is not always straightforward. One of the challenges in this context is to develop techniques that can robustly deal with growing objects, changing appearance, the development of new organs causing changes in the topology as well as non-rigid deformations caused by external factors such as sunlight, wind, gravity, etc. This is illustrated in Fig 1 where we perceive these large changes in the time-series data of two plant types which were recorded with a laser scanner recorded over a period of two weeks. Typically, point cloud registration is performed using iterative closest point-based approaches [8]. These approaches, however, are often unable to capture the deformations in the object and are prone to divergence due to new or missing plant organs. In this paper, we investigate the means to account for the growth and the non-rigid deformations that the plants undergo.

The main contribution of this paper is a fully automatic registration technique for plant point clouds which have been acquired over time. We propose to use the skeleton structure of the plant to drive the registration process as it provides a compact representation of the plant by capturing its overall shape and topology. In this paper, we propose a method for extracting the skeletal structure along with semantic information to perform the data association step. We classify each point of the plant as a leaf or stem point, a further clustering allows us to compute individual leaf instances. We then determine correspondences between plant skeletons using a hidden Markov model. These correspondences in turn allow us to estimate parameters, which are able to capture the deformation and the growth of the plant skeleton. We then transfer the deformations estimated on the plant skeletons to the whole point cloud to register the temporally separated point clouds. Using these registration parameters, we are also able to interpolate over the registration parameters to obtain an estimated point cloud at a time instant in-between the actual acquisition times.

In sum, our approach is able to (i) register temporally separated plant point clouds by explicitly accounting for the growth, deformations, and changes in the plant topology, (ii) exploit the skeletal structure as well as semantic information computed from the data, (iii) find correspondences between the different organs of the plant and track plant growth parameters over time, (iv) demonstrate reliable registration results on long-term datasets of two types of plants captured using a 3D laser scanner mounted on a robotic arm.

## Related work

Phenotyping plays an important role in plant sciences. Traditionally, a lot of the activities required substantial manual work. Over the last decade, however, automated plant phenotyping has been receiving increasing interest, also in other disciplines such as robotics [10, 11] or computer vision [12–14]. One relevant aspect in this context relates to obtaining relevant features of plants, often referred to as phenotypic traits, in an automated manner [2, 15]. Several phenotyping systems [1, 16, 17] have been developed for greenhouse environments and are also available commercially [18, 19]. Other systems such as [20–24] have been designed to acquire plant data directly from the fields or estimate information about plants for management actions [25–28].

These phenotyping systems have been used for several applications such as plant growth monitoring [5], drought stress analysis [29], canopy cover estimation [30], horticulture [31, 32] etc. Several approaches [33–37] aim at obtaining traits at a coarse resolution over the entire field using image data captured from UAVs as well as from ground robots. More recently, 3D laser data has been used in many agricultural applications such as [38–40] and analyzed to obtain phenotypic traits with high fidelity [5, 6, 41]. Li *et al*. [42] and Paproki *et al*. [43] analyze time-series point cloud data to detect topological events such as branching, decay and track the growth of different organs. While both works emphasize obtaining phenotypic traits at an organ level, our focus in this paper is on developing basic techniques for matching as well registering temporally separated scans of individual plants using the whole point cloud data. This technique forms the basis for tracking phenotypic traits over time.

Several approaches in the past have attempted to leverage the topological structure of the object to drive the registration process, primarily in the field of human motion tracking [44–46]. A large number of techniques exist for extracting skeletons from 3D models, also known as skeletonization. The skeleton structure of the model is then used for different applications such as animation, tracking, surface reconstruction, etc. Huang *et al*. [47] use local L1-medial points to compute the skeleton curve for an unorganized point cloud, while Tagliasacchi *et al*. [48] propose the idea of a generalized rotational symmetry axis of the model which exploits the normal information as well. Recently, Wu *et al*. [49] proposed a technique using Laplacian point cloud contraction to extract skeletons of maize point clouds. A detailed state-of-the-art review for extracting skeletons of 3D objects is given in [50]. In contrast to these approaches, we exploit both supervised and unsupervised machine learning techniques to compute the skeleton curve for the plant point clouds. In this process, we classify the plant into stem and leaf points, cluster them together as individual organs and use this semantic information for computing the skeleton structure effectively.

Exploiting semantic information of the plant for extracting the skeleton structure is quite helpful. While a large corpus of literature exists for classification in 2D images, the number of approaches that operate on 3D point clouds is rather small. Paulus *et al*. [51] propose an SVM-based classifier that relies on a surface histogram to classify each point in a 3D point cloud as leaf or stem. The recent approach by Zermas *et al*. [52]use an iterative algorithm called randomly intercepted node to tackle the same problem. Sodhi *et al*. [53] use 2D images to extract 3D phytomers, namely fragments of the stem attached to a leaf for leaves detection. Shi *et al*. [54] propose a multi-view deep learning approach inspired by Su *et al*. [55] to address the organ segmentation problem, while Zermas *et al*. [56] uses a skeletonization approach to segment leaf instances. Our approach builds upon the work of Paulus *et al*. [51] and additionally groups the leave points with an unsupervised clustering algorithm to extract leaf instances. In this way, we achieve an organ segmentation exploiting labeled data for the leaves. We use this in turn as the basis for our registration approach across plant point clouds.

Recent developments for segmenting point cloud data using deep-learning techniques are promising. Several methods have been proposed to segment point clouds, among which PointNet [57]and PointNet++ [58] are often considered as milestones. Several architectures using special convolution operations designed for point clouds such as KPConv [59]provide state-of-the art segmentation results. However, a common requirement for many such techniques is the availability of large-scale labeled datasets. In our application where we are using high resolution plant point cloud data, training datasets at such scale are not available as of yet. Moreover, in this work we essentially use the segmentation results to generate a skeleton from the point-cloud. Since, all other steps in the registration process are designed to cope with imperfections in the skeleton structure, having a precise segmentation for the point cloud is not critical for this approach. As a result, we did not investigate the use of 3D deep learning networks in this work. These techniques can form the basis for segmentation task as opposed to the use SVM as done in this work.

Registering point clouds is a common problem in a lot of disciplines and multiple techniques have been proposed for laser-based or RGB-D-based mapping systems [8, 9, 60]. These techniques work under the assumption the objects being registered only undergo rigid motion. The techniques have been extended by relaxing the rigidity assumption and several non-rigid registration techniques such as [61–63] aim at capturing the deformation in the object. Approaches such as [64–68] aim at reconstructing scenes in an online fashion either in the presence of dynamic objects or deformations. Such approaches typically operate on scans captured at a high frame rate (10-30 Hz) and thereby deal with rather small deformations in between consecutive scans. This assumption is violated for the point cloud data considered here. In our application, the plants are usually scanned at a comparably low frequency (once per day) and thereby showing larger growth and deformations in between consecutive scans. In addition, the problem becomes even more difficult if the object changes its appearance and topology over time. Zheng *et al*. [69] propose an approach to register 3D temporal point clouds of objects by exploiting skeleton structures. In this paper, we build upon their formulation and integrate it within an improved data association approach which accounts for the topology and the semantic of the plant. We then use these data associations to register temporally separated 3D plant point clouds within an iterative non-rigid registration scheme.

This paper extends our previous conference paper [70] in several ways. First, the prior work required a manually provided skeletonization of the plants, while in this paper, we estimate it from sensor data. Second, we estimate semantic information about the plant organs and exploit this estimate to compute a high-quality skeleton and improve the data association between point clouds. Furthermore, we substantially extended the discussion of related work and provided additional experiments for maize in addition to tomato plants. All these claims are backed up through the experimental results presented in this paper. The implementation of our approach is available at: https://github.com/PRBonn/4d_plant_registration.

## Material and methods

### Proposed approach to plant point cloud registration

Our approach operates on a time-series of 3D point clouds of plants. Our registration procedure starts with extracting a skeleton along with the organ level semantics for each point cloud. The skeletons are undirected acyclic graphs, which represent the topology or the inner structure of the plant. Each node contains the *x*, *y*, *z* coordinates of its position, a 4x4 affine transformation matrix *T* to describe the local transformation, and a semantic class label as attributes. The skeletons are extracted from the point cloud data and are often imperfect. This fact is taken into consideration during the registration procedure. We also operate directly on the ordered point clouds and do not require a mesh structure or other cues such as the normals providing the inside-outside information of the surface. The skeleton structures allow us to compute data associations between temporally separated 3D scans and use these correspondences to perform an iterative procedure which registers the plant scans obtained at different times. Finally, the registered skeletons can be used to deform the complete point cloud, e.g., the point cloud from time step *t*_1_ deformed to time step *t*_2_. The overall registration scheme is summarized in Alg. 1 and will be explained in detail in the following sections.

**Algorithm 1** Skeleton-driven iterative non-rigid registration procedure

1: $P_{1},P_{2}$ ⊳ Input point clouds

2: $C_{12}^{t-1},C_{12}^{t}=\emptyset$ ⊳ Initialization

3: $O_{1}\leftarrow \text{performInstanceSegmentation}(P_{1})$ ⊳ Segment $P_{1}$

4: $O_{2}\leftarrow \text{performInstanceSegmentation}(P_{2})$ ⊳ Segment $P_{2}$

5: $S_{1}\leftarrow \text{computeSemanticSkeleton}(P_{1},O_{1})$ ⊳ Compute skeleton $S_{1}$

6: $S_{2}\leftarrow \text{computeSemanticSkeleton}(P_{2},O_{2})$ ⊳ Compute skeleton $S_{2}$

7: **while** $\left(C_{12}^{t}\backslash C_{12}^{t-1}\right)\cup \left(C_{12}^{t-1}\backslash C_{12}^{t}\right)=\emptyset$ **do** ⊳ Repeat until matches are same

8:  $C_{12}^{t-1}=C_{12}^{t}$

9:  $C_{12}^{t}\leftarrow \text{findSkeletalCorrespondences}(S_{1},S_{2})$ ⊳ Compute matches

10:  $T_{12}\leftarrow \text{compSkeletalDeformation}(S_{1},S_{2},C_{12}^{t})$ ⊳ Compute deformation

11: $\hat{P_{1}}\leftarrow \text{applyDeformation}(P_{1},T_{12})$ ⊳ Apply deformation to $P_{1}$

### Extracting the skeletal structure along with semantics

The first step of our approach is to extract the skeleton structure S of the plant from the input point cloud P. To achieve such goal, we first perform a segmentation step aiming at grouping together points belonging to the same plant organ, namely one leaf instance or the stem. To do this, we start by classifying each point of the point cloud P as a point belonging to either the stem or leaf category. We use a standard support vector machine classifier with the *x*, *y*, *z* coordinates along with with the fast point feature histograms [71] as a feature vector. The fast point feature histograms technique computes a histogram of directions around a point using the neighborhood information, thereby capturing the local surface properties in a compact form. With these feature vectors as inputs, the support vector machine can classify each point of a plant point cloud into stem and leaf points. We train the support vector machine model using the scikit-learn library [72] in a supervised manner by providing labels for two randomly sampled scans of the temporal sequence as the training set.

After the model is trained, we use it to predict the semantics (category) for all the remaining point clouds of the sequence. Once the classification step is complete, we perform a clustering step in order to find the individual leaves or the stem as unique instances. We perform the clustering using the density-based spatial clustering algorithm [73]. It uses the *x*, *y*, *z* coordinates of the points to obtain an initial segmentation, which is then refined by discarding small clusters and assigning each discarded point to one of the remaining clusters based on a *k*-nearest neighbor approach.

**Fig 2:**
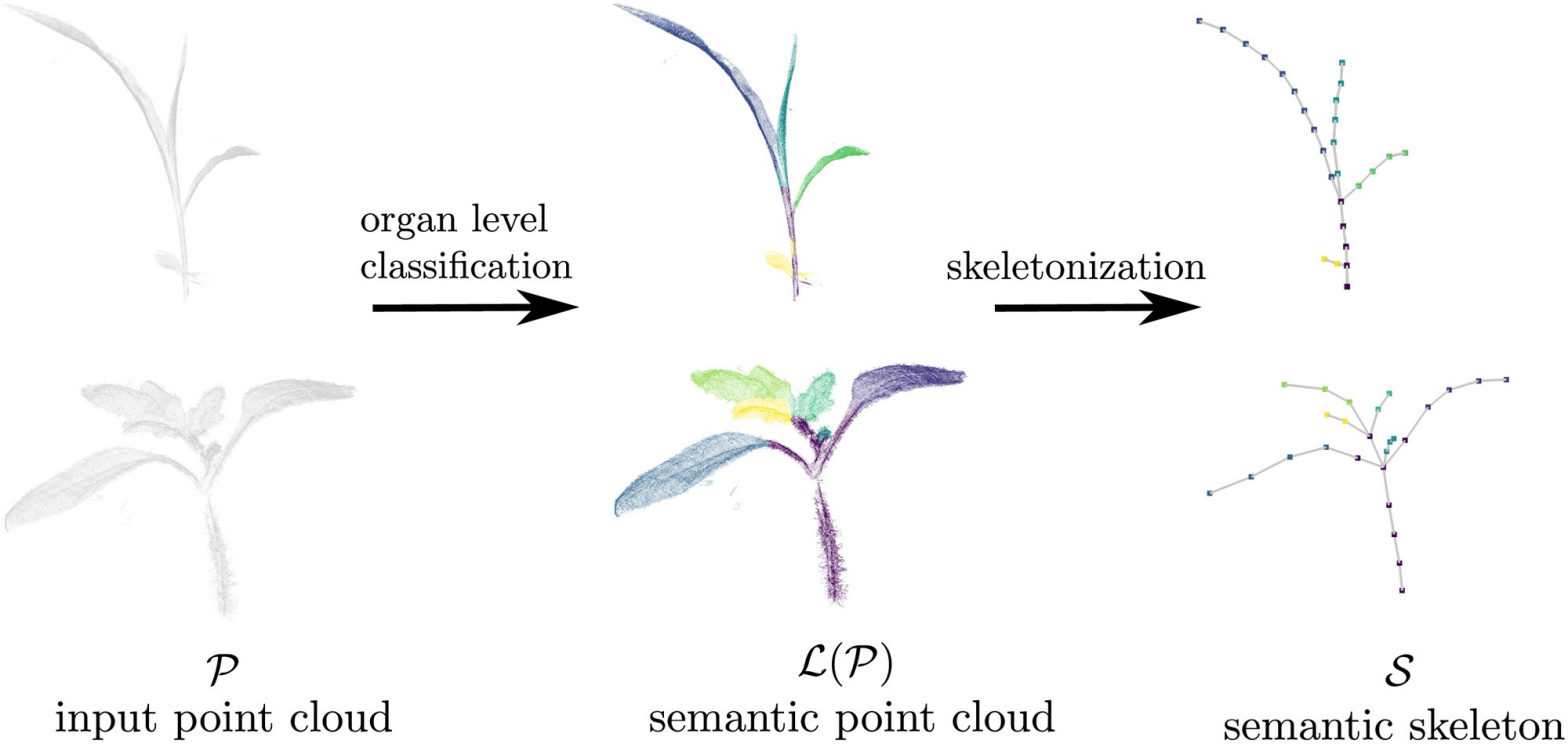
Extracting skeletal structure for using semantics of the plant. The figure illustrates the skeletonization pipeline for a maize (top) and tomato (bottom) plant scan. Note that for the tomato plant, we classify individual leaflets (green + yellow + light-blue) as separate instances rather than as an individual leaf. The leaflets can be combined into a single leaf in case this distinction is not desired/required for the application.

At this stage, each point in the point cloud is assigned to an organ, namely to the stem or to one leaf instance. We then learn keypoints for each organ using self-organizing maps [74]. These keypoints would form the nodes of the skeleton structure. Self organizing maps are unsupervised neural networks using the concept of competitive learning instead of back-propagation. They take as input a grid that organizes itself to capture the topology of the input data. Given an input grid G and the input set of points P, the self organizing map defines a fully-connected layer between G and P. This network learns a transformation the grid G points in manner to cluster the data P effectively. The learning process is composed of two alternating steps until convergence. First, the winning unit is computed as:

$$\begin{array}{c} x=\underset{p\in P}{\text{argmin}}∥p-w_{i}∥, \end{array}$$ (1)

where *p* is a randomly chosen sample from P and *w*_*i*_ is the weight vector most similar to *x*, also called the best matching unit. The second step consists of updating the weights of each unit according to:

$$\begin{array}{c} w_{n}=w_{n}+\eta \;\beta \left(i\right)\;\left(x-w_{i}\right), \end{array}$$ (2)

where *η* is the learning rate and *β*(*i*) a function, which weights the distance between unit *n* and the best matching unit. In our case, the grid for each organ is an *n* × 1 chain of 3D points that will form the nodes along the skeleton for that organ. The length of the chain *n* is proportional to the size of the organ, such that the keypoints are expected to have a minimum distance between 1 cm between them. In this way, it is possible to obtain a skeleton-like structure for each plant of the temporal sequence of plant point clouds. Fig 2 visualizes the organ segmentation as well as the skeleton structures extracted from the input point cloud P for two sample scans of our dataset.

### Estimating skeletal correspondences

Before data from any 3D objects can be aligned, we need to establish the data associations between the sensor readings, i.e, estimating which part of cloud $P_{1}$ corresponds to which parts of cloud $P_{2}$. Establishing this data association is especially hard for objects that change their appearance and automatic processes are likely to contain data association outliers, which will affect the subsequent alignment. For registering temporally separated plant scans, we propose to obtain these data associations by matching the corresponding skeleton structures and not work directly on the raw point clouds.

In this data association step, we estimate correspondences between two skeletons by exploiting their geometric structure and semantics, which are computed using the approach described in the previous section. As the skeleton structure and the semantics are estimated from sensor measurements, it might suffer from several imperfections. To cope with these imperfections in the individual skeletons and inconsistencies between them, we use a probabilistic approach to associate the skeleton parts as opposed to graph matching approaches, which typically do not tolerate such errors well. We, therefore, formulate the problem of finding correspondences between the skeleton pair ($S_{1}$,$S_{2}$) using a hidden Markov model formulation [75]. The HMM model provides the flexibility to encode different cues, define constraints for the correspondences, as well as include prior information about the skeleton structure. This allows us to track several correspondence candidates and choose the best correspondences between the skeleton pair.

The unknowns or the hidden states of the HMM model represent all the potential correspondences between the nodes of the two skeletons. In addition, we also add a so-called “not matched state” for each node to the HMM to account for the situations in which the node may have no correspondences at all. This happens, for example, when nodes belong to new organs that were not present before or when new nodes emerge on the curve skeleton due to the plant growth. As required in a HMM formulation, we need to define the emission cost *Z* and the transition cost Γ. The emission cost *Z* describes the cost for a given hidden state (correspondence) to produce a certain observation. In our case, the observations are the sequence of nodes of the first skeleton $S_{1}$ arranged in depth first manner starting from the node at the base of the stem. We define this cost for a correspondence $c_{ij}\in C_{12}$ between node *n*_*i*_ of $S_{1}$ and node *n*_*j*_ of $S_{2}$ as:

$$\begin{array}{ccc} Z(c_{ij}) & = & w_{d}\;|deg\left(n_{i}\right)-deg\left(n_{j}\right)|+w_{e}\;∥x_{i}-x_{j}∥+w_{sem}\;\rho_{sem}\left(L\left(n_{i}\right),L\left(n_{j}\right)\right), \end{array}$$ (3)

where we the first term yields the absolute difference (denoted by |⋅|) between the degrees of the corresponding nodes, where *deg*(*n*) is the number of edges incident to a node. The second term refers to the Euclidean distance (denoted by ‖⋅‖) between them with *x*_*i*_, *x*_*j*_ being the 3D locations of the nodes *n*_*i*_, *n*_*j*_ respectively. The final term *ρ*_*sem*_ is one in case the semantics for the corresponding nodes $L\left(n_{i}\right),L\left(n_{j}\right)$ the are not the same, otherwise, it is set to zero. The idea behind combining these three terms is to capture the geometric aspects, i.e., the topology difference, the spatial distance between the nodes, and the semantics of the skeleton nodes being matched. This combined cost will be smaller for correspondences between nodes that have similar topology, are located close to each other, and have the same semantic label. We weigh all the three terms using *w*_*d*_, *w*_*e*_, and *w*_*sem*_ to scale the measures such that they are in a similar range. We set *w*_*d*_ = 1, *w*_*e*_ = 10 and *w*_*sem*_ = 1 for all of the experiments.

The transition cost Γ describes the cost involved in transitioning from one hidden state *c*_*ij*_ to another *c*_*kh*_. This can be treated as the cost involved in having *c*_*kh*_ as a valid match given that *c*_*ij*_ is a valid match as well. We define this cost as:

$$\begin{array}{ccc} \Gamma (c_{ij},c_{kh}) & = & |d_{g}\left(n_{i},n_{k}\right)-d_{g}\left(n_{j},n_{h}\right)|\\ & & +w_{nbr}\;\left|n_{br}\left(n_{i},n_{k}\right)-n_{br}\left(n_{j},n_{h}\right)\right|\\ & & +\rho_{dir}\left(\left(x_{i}-x_{j}\right),\left(x_{k}-x_{h}\right)\right), \end{array}$$ (4)

where the first term computes the difference of the geodetic distances *d*_*g*_ between the nodes involved in the two correspondence pairs along their respective skeletons. This means that a pair of correspondences (*c*_*ij*_, *c*_*kh*_) having equal geodetic lengths *d*_*g*_(*n*_*i*_, *n*_*k*_) along $S_{1}$ and *d*_*g*_(*n*_*j*_, *n*_*h*_) along $S_{2}$ will have a lower cost than the ones which have much different lengths along the skeleton. The second term captures the difference in the number of branches *n*_*br*_, i.e., nodes with degree greater than 2, along the way on the skeleton. The weight *w*_*nbr*_ is automatically set as the maximal geodetic distance between all node pairs of the first skeleton. The final term *ρ*_*dir*_ is a function that penalizes the correspondence pairs (*c*_*ij*_, *c*_*kh*_) with a large cost if the directions determined by (*x*_*i*_ − *x*_*j*_) and (*x*_*k*_ − *x*_*h*_) are opposite, i.e., the angle between them are greater than $\frac{\pi }{2}$. We set the penalty cost as the maximum geodesic distance between any two nodes on $S_{1}$.

**Fig 3:**
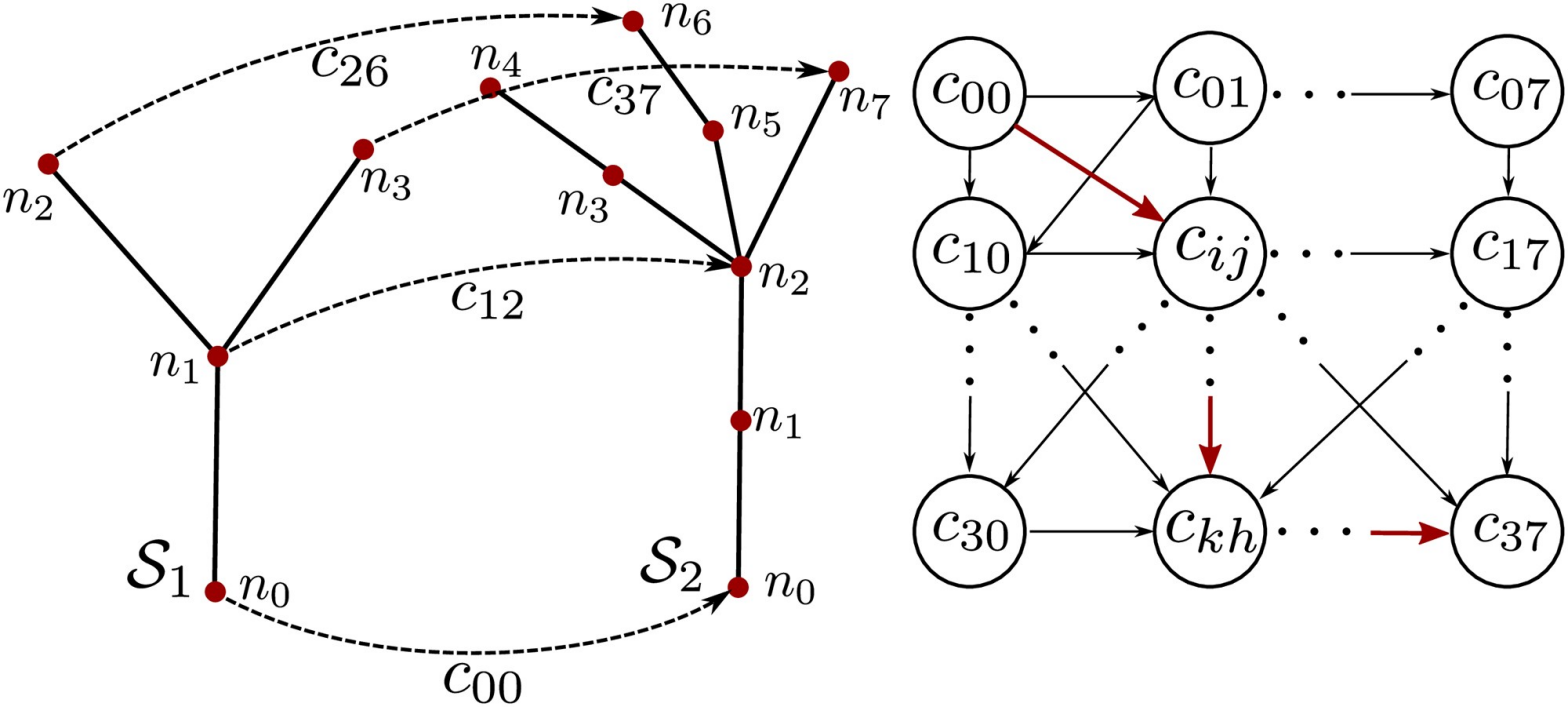
Left: Skeletal matching for an example pair of plant point clouds with the variables involved. Right: Hidden Markov model (HMM) used for correspondence estimation. We only show a subset of the hidden variables, i.e. the potential correspondences, in the HMM. The red line depicts the sequence of best correspondence estimated by the Viterbi algorithm. This produces the correspondences between $S_{1}$ and $S_{2}$ visualized with the dash-lined arrows on the left.

Once the emission and transition costs are defined, we compute the correspondences between the skeletons by performing inference on the HMM. The result is the most likely sequence of hidden variables, i.e., the set of correspondences between $S_{1}$ and $S_{2}$. We perform this inference using the Viterbi algorithm [76]. In case a node has more than one correspondence, we choose the correspondence with the smaller Euclidean distance to ensure a one-to-one correspondence. As an illustration, Fig 3 (left) shows an example skeleton pair for which we want to estimate the correspondences $C_{12}$. Fig 3 (right) depicts the HMM for the example pair where the red path indicates the set of correspondences estimated by the Viterbi algorithm. The HMM model only shows a subset of the connections between the hidden states, where in practice each state is connected to every other state.

### Computing skeletal deformation parameters

In this step, we compute the registration parameters between $S_{1}$ and $S_{2}$ given the set of correspondences $C_{12}$. While registering temporally separated plant scans, we need to take into account the plant growth, which manifests in the form of change in shape and the topology of the plant. Therefore, to capture these changes we need to forego the usual assumption of rigidity, often used in point cloud registration [8]. Our goal is to capture the non-rigid changes by computing sets of deformation parameters between skeleton parts of the respective plant scans. We estimate these deformation parameters through a non-linear least-squares optimization procedure based on the correspondences obtained from the procedure described in the previous section.

**Fig 4:**
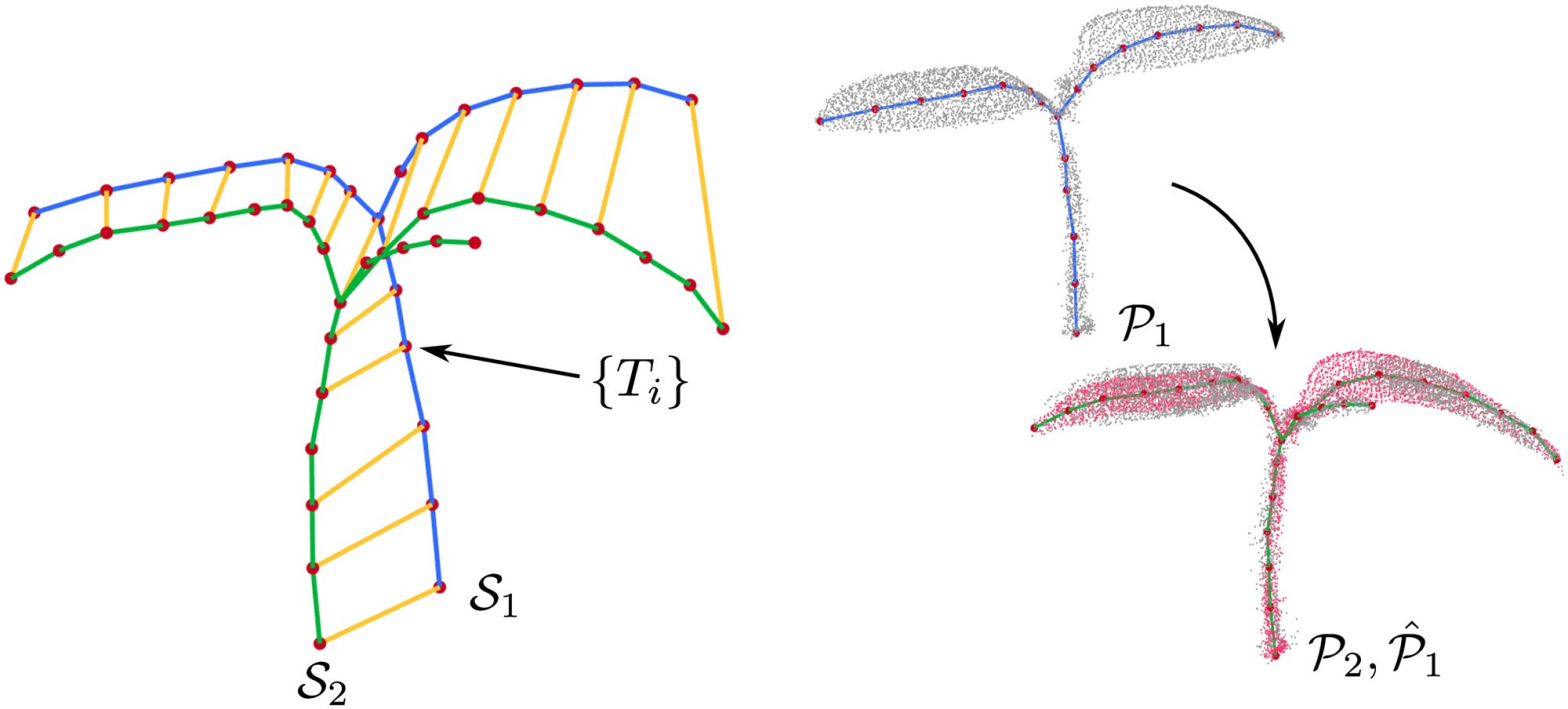
Left: Registering the skeleton pair involves estimating the deformation parameters attached to the nodes of the source skeleton $S_{1}$. Right: Transferring the deformation results to the entire point cloud.

To model the deformations between the plant scans, we attach an affine transformation *T*_*i*_ to each node *n*_*i*_ of the skeleton $S_{1}$ as illustrated in Fig 4 (left). The intuition behind such a model is that the skeleton may be deformed differently at different locations along the skeleton. By modeling the deformations through a 3D affine transformation with 12 unknown parameters per node, we are able to capture the growth as well as bending of the plant via the scaling, shearing, and rotation parameters.

We define the objective function of the optimization problem as a combination of the three energy terms. The first term *E*_*corresp*_ is defined as:

$$\begin{array}{ccc} E_{\text{corresp}} & = & \sum_{c_{ij}\in C_{12}}∥T_{i}\;x_{i}-y_{j}∥, \end{array}$$ (5)

where *x*_*i*_ and *y*_*j*_ are the node positions given by the correspondence pair *c*_*ij*_ obtained using the procedure described in the previous section. This energy term captures the distance between corresponding nodes in $S_{1}$ and $S_{2}$ for an estimate of the affine transformation *T*_*i*_. The goal of the optimization procedure would be to choose such a transformation *T*_*i*_ attached to each node *n*_*i*_ which makes the overall error in Eq (5) as small as possible.

The second energy term *E*_*rot*_ captures how close the estimated affine transformation is to a pure rotation and it determines the smoothness of the deformation. We define *E*_*rot*_ as:

$$\begin{array}{ccc} E_{\text{rot}} & = & \sum_{\begin{array}{c} i=1\\ j=\text{mod}(i+1,3) \end{array}}^{3}{\left(c_{i}\;c_{j}\right)}^{2}+\sum_{i=1}^{3}{\left(c_{i}\;c_{i}-1\right)}^{2}, \end{array}$$ (6)

where *c*_*i*_ represents the columns of the rotation part of affine transformation, i.e., the first three rows and columns of *T*_*i*_). The first term in *E*_*rot*_ in Eq (6) measures the deviation for a pair of columns to be orthogonal with each other, whereas the second term measures the deviation of each column from being unit length. *E*_*rot*_ forces the estimated affine parameters *T*_*i*_ to be as close to a true rotation as possible. This energy terms keeps the shearing effect in check and results in naturally looking deformations.

We also define a regularization term *E*_*reg*_ as:

$$\begin{array}{ccc} E_{\text{reg}} & = & \sum_{j\in N(i)}{\text{norm}}_{F}\left(T_{i}^{-1}\;T_{j}-I\right), \end{array}$$ (7)

where *T*_*i*_, *T*_*j*_ are transformations corresponding to nodes *n*_*i*_, *n*_*j*_ such that *j* is the neighbor *N*(*i*) along $S_{1}$, and norm_*F*_ is the Frobenius norm after performing the homogeneous normalization of the involved matrices. *E*_*reg*_ is a regularizing term, which forces the transformation parameters of neighboring nodes to be similar. This results in a smooth deformation along the skeleton and achieves similar results as the as-rigid-as-possible constraint described by Sorkine *et al*. [62]. The regularization term is also necessary to constrain the nodes that do not have any correspondences. Finally, the combined energy *E*_*total*_ is obtained as a weighted combination of all the three energies as:

$$\begin{array}{ccc} E_{\text{total}} & = & w_{\text{corresp}}\;E_{\text{corresp}}+w_{\text{rot}}\;E_{\text{rot}}+w_{\text{reg}}\;E_{\text{reg}} \end{array}$$ (8)

We use the weights *w*_*corresp*_ = 100, *w*_*rot*_ = 10, and *w*_*reg*_ = 1 for all the example in our datasets. The weights have been chosen such that the cost due to each component of the loss is in the same order of magnitude. We employ the Gauss-Newton algorithm to solve the unconstrained non-linear least squares problem [77]. We use the Cauchy robust kernel [78] for the error residuals belonging to *E*_*corresp*_ as this prevents incorrect correspondences to influence the optimization process. The robust kernel automatically downweights potentially wrong correspondences, which have large residuals. The approach is related to the formulation by Sumner *et al*. [63] for estimating deformation parameters for surfaces parametrized as triangular meshes. In our case, we adapt the energy terms to reflect the constraints valid for deformation between curve skeletons as opposed to surfaces. Furthermore, the approach by Sumner *et al*. [63] is not able to fully constrain the nodes which have a degree smaller than 3, but is essential for registration of curve skeletons.

### Point cloud deformation

Traditional approaches to point cloud registration assume rigid objects. In this case, the alignment results in the execution of a 6 degree of freedom transformation consisting of rotations and translations. This, however, is substantially different in our case. To obtain the final registered point cloud ${\hat{P}}_{1}$ of a growing plant, we need to apply the deformation parameters estimated for the skeleton nodes to all the 3D points of the scan. This means, that the individual data points will be affected by individual affine transformation to obtain the aligned cloud. An example of the point cloud deformation is visualized in Fig 4 (right).

For each point $p\in P_{1}$, we obtain the deformed point $\hat{p}$ as a weighted sum of affine transformations corresponding to the two nearest nodes to the point *p* as

$$\begin{array}{ccc} \hat{p} & = & \sum_{k\in N(p)}\alpha_{k}\;T_{k}\;p, \end{array}$$ (9)

where *k* is the index of the nearest node *N*(*p*) and *α*_*k*_ is computed according to the projection of the point *p* on the edge of the skeleton determined by the nearest nodes. Let *p*_*e*_ be the projection of point *p* on edge *e*. Then the weight is given by:

$$\begin{array}{ccc} \alpha_{k} & = & 1-\frac{∥p-e∥}{∥e∥}. \end{array}$$ (10)

### Iterative non-rigid registration procedure

We use the steps from the previous sections to formulate an iterative approach to register the point cloud pair $\left(P_{1},P_{2}\right)$ as summarized in Alg. 1. Similar to the popular ICP approach [8], we alternate between correspondence estimation steps and registration steps given the correspondences. We start out by computing the organ level instance segmentation and skeleton structure with semantic information (lines 3-6 of Alg. 1). We then start the iterative procedure, which alternates between estimating the correspondences $C_{12}$ (line 9) and the registration parameters, i.e., the 3D affine transformations attached to each node (line 10). By iterating through these steps multiple times, we can obtain new correspondences, which might not have been captured due to the large distance between the skeletons given their initial configuration. Finally, we exit the iterative scheme when there is no change in the estimated correspondence set $C_{12}^{t}$. After computing the registration parameters $T_{12}$ between the nodes of the skeletons $S_{1}$ and $S_{2}$, we apply these parameters to the entire point cloud $P_{1}$, which results in the registered point cloud $\hat{P_{1}}$ (line 11).

### Interpolating point clouds of plants

In addition to registering the plant scans recorded at different times, we would also like to interpolate how the plant may be deformed at an intermediate time in between the actual acquisition times. This may allow for increasing the time between the recordings and doing further analysis on the interpolated point clouds. We compute the deformed point cloud by interpolating the deformation parameters *T* estimated between the two registered scans. To obtain a smooth interpolation, we first decompose of the estimated affine transformation *T* into scale/shear transformation *T*_*s*_, pure rotation *T*_*R*_, and a pure translation *T*_*t*_ using the polar decomposition approach described by Shoemake [79] and is given by:

$$\begin{array}{ccc} T & = & T_{s}\;T_{R}\;T_{t}. \end{array}$$ (11)

We then linearly interpolate *T*_*s*_ and *T*_*t*_ to obtain the transformation at time t. For interpolating *T*_*R*_, we use the spherical linear interpolation described by Shoemake [80].

### Computing phenotypic traits

In this work, we compute different phenotypic traits for both the stem and leaf instances. In particular, for the stem class, we compute stem diameter and stem length, whereas for the leaf class, we compute the leaf length and area. We choose these traits as examples, and other geometric traits that use the area or shape of the organs can be computed and tracked over time as well.

The stem and leaf length can be easily computed from the geometric information of the semantic skeleton: The stem/leaf length is just the sum of the lengths of the edges of all nodes of a particular instance.

For computing the stem diameter *s*_*d*_, we first assign each point in the point cloud classified as stem to the closest node on the skeleton. Then, we compute the main axis of the data distribution in the neighboring region of the selected node using a standard singular value decomposition (SVD) approach. We can then compute the stem diameter with respect to the main axis of the considered neighborhood. The stem diameter *s*_*d*_ is obtained as:

$$\begin{array}{ccc} s_{d} & = & 2\sum_{n}\frac{1}{k_{n}}\sum_{k_{n}}∥p_{k}-\pi_{l}\left(p_{k}\right)∥, \end{array}$$ (12)

where *n* is the number of nodes of the stem, *k*_*n*_ is the number of points in the point cloud assigned to the *n*-th node, and *π*_*l*_(⋅) is a function which projects a point on the main axis.

We compute the leaf area *l*_*a*_ by exploiting the SVD to estimate the main plane *A* of the points associated with each node. This gives us the leaf area *l*_*a*_ as:

$$\begin{array}{ccc} l_{a} & = & \sum_{m}\text{hull}\left(\pi_{A}\left(p_{n}\right)\right), \end{array}$$ (13)

where hull(⋅) represents the area of the convex hull and *π*_*A*_(⋅) the projection of all points *p*_*n*_ associated with the *n*^th^ node on the main plane *A*. The final sum is taken over all *m* nodes of a leaf.

## Experimental results

### Dataset description

We evaluate our approach on time-series 3D point cloud data of three sample plants of maize (*Zea mays*) and tomato plants (*Solanum lycopersicum*). The scans were recorded using a robotic arm (Romer Absolute Arm) [81] equipped with a high precision laser scanner. The dataset was recorded daily over a period of 10 days. The plants have been scanned in manner so as to minimize self occlusions whenever possible. The point cloud data undergoes a pre-processing step where all the points which do not belong to the plant are removed, for example the points belonging to the soil and the pot. The datasets cover substantial growth of the plants, starting from the 2 cotyledons (i.e. seed leaves) at the start to around 8 leaflets (2 cotyledons + 2 leaves) for the tomato plants, and 1 to 4 leaves for the maize plants. The plants undergo substantial leaf and stem growth, and includes several branching events till the end of the dataset acquisition period as illustrated in Fig 1. The height of the tomato plants reaches up to 150 mm and the maize plant reaches over 400 mm. The data used in the paper is available at: https://www.ipb.uni-bonn.de/data/4d-plant-registration/.

### Semantic classification of plant point clouds

**Fig 5:**
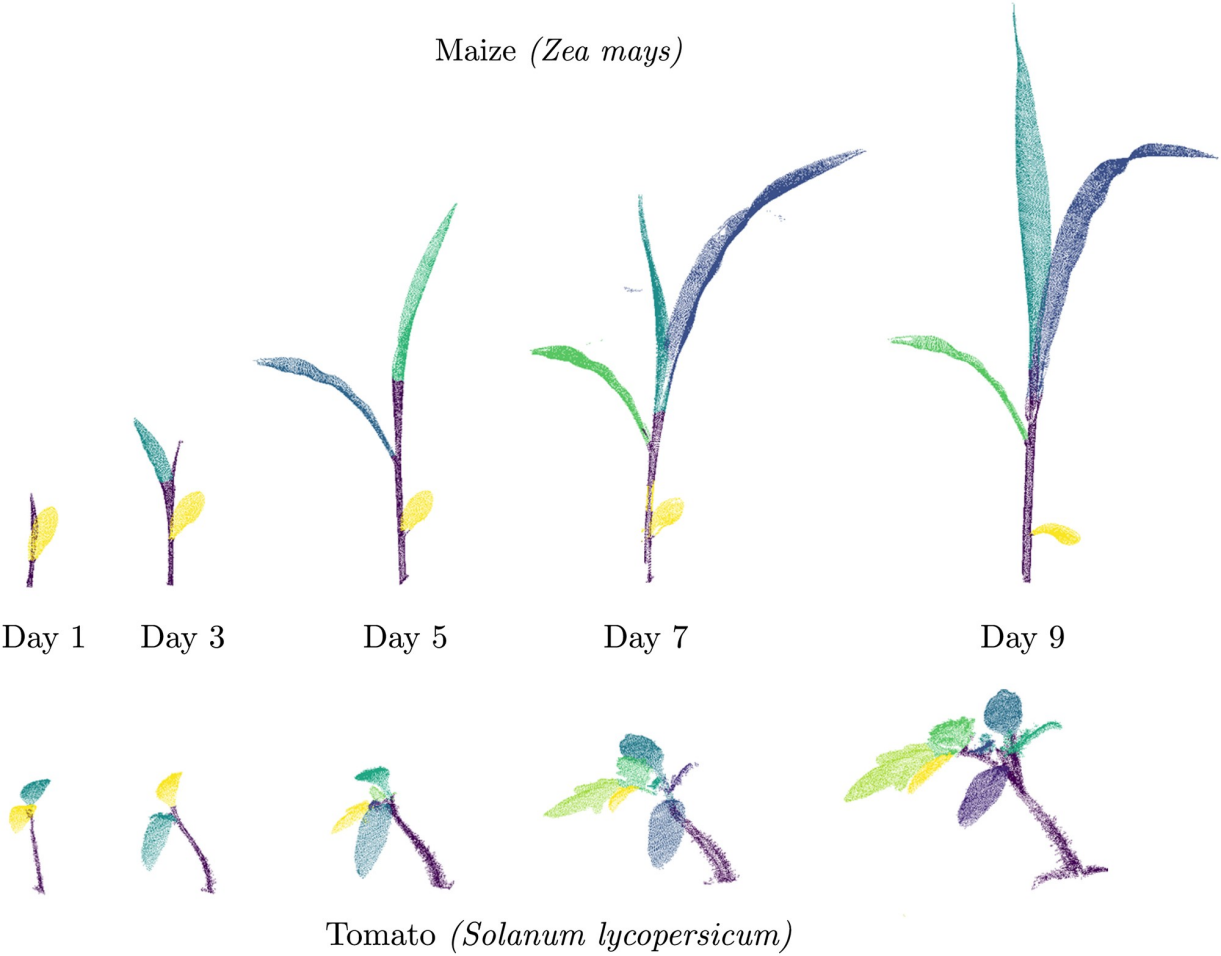
Semantic classification of maize (top) and tomato (bottom) point clouds. Each stem and leaf (or leaflet) instance is visualized with a different color. Note that the colors of same leaf instances do not correspond over time, as data associations have not been computed at this stage.

In the first experiment, we evaluate the performance of our approach for organ level semantic classification. The classification system has been trained on two randomly selected point clouds from each datasets. All the remaining point clouds in the sequence are used as test datasets. The ground truth information for both the training and test sets have been generated manually by human users. We show the qualitative results computed by our approach by visualizing the semantic instances for some point clouds from the two datasets in Fig 5. Each stem and leaf instance is visualized with a different color. We can visually inspect the stem and the leave instances throughout the temporal sequence, and see that the classification is successful for instances despite their size and shape changing over time. The colors of same leaf instances do not persist over the temporal sequence since the data associations between them have not been computed at this stage.

We also perform a quantitative evaluation of the classification performance of our classification approach by computing standard metrics [82] such as precision $p=\frac{\text{tp}}{\text{tp}+\text{fp}}$ (Table 1), recall $r=\frac{\text{tp}}{\text{tp}+\text{fn}}$ (Table 2), and intersection over union (IoU) $IoU=\frac{\text{tp}}{\text{tp}+\text{fp}+\text{fn}}$ (Table 3), for the maize and tomato plant datasets. For each metrics, we show the minimum and maximum values over the dataset as well as the standard deviation. In the definitions above, *tp* stands for true positive, *fp* for false positive, and *fn* for false negative. In all tables, the SVM is responsible for the stem and the leaf class, while instance refers to the unsupervised clustering of individual leaves. We obtain over 90% precision and recall for leaf point in both the datasets, whereas they are around 85% for the stem points. Regarding the leaves instances, both metrics are around 90%.

**Table 1:** Precision values for class-wise and instance segmentation on our datasets.

Dataset	Stem				Leaf				Instances			
	mean	min	max	std	mean	min	max	std	mean	min	max	std
Maize	86.3	74.5	99.6	8.6	95.5	93.6	99.4	2.2	94.4	91.9	99.6	2.7
Tomato	89.6	68.6	99.2	9.6	97.9	96.9	99.0	0.9	83.4	59.8	99.7	15.4

**Table 2:** Recall values for class-wise and instance segmentation on our datasets.

Dataset	Stem				Leaf				Instances			
	mean	min	max	std	mean	min	max	std	mean	min	max	std
Maize	85.7	48.6	99.1	16.3	92.9	89.1	99.8	3.3	94.7	91.3	99.6	3.1
Tomato	92.2	60.6	99.4	11.4	96.5	74.0	99.6	8.3	78.2	66.6	99.4	11.2

**Table 3:** Intersection over union (IoU) score for our datasets.

Dataset	Stem				Leaf				Instances			
	mean	min	max	std	mean	min	max	std	mean	min	max	std
Maize	80.0	47.8	94.6	12.7	94,6	88.9	97.4	2.5	94.0	91.0	97.6	2.3
Tomato	82.6	60.6	92.2	10.4	93.4	73.9	99.0	7.8	69.1	51.4	98.7	15.7

**Fig 6:**
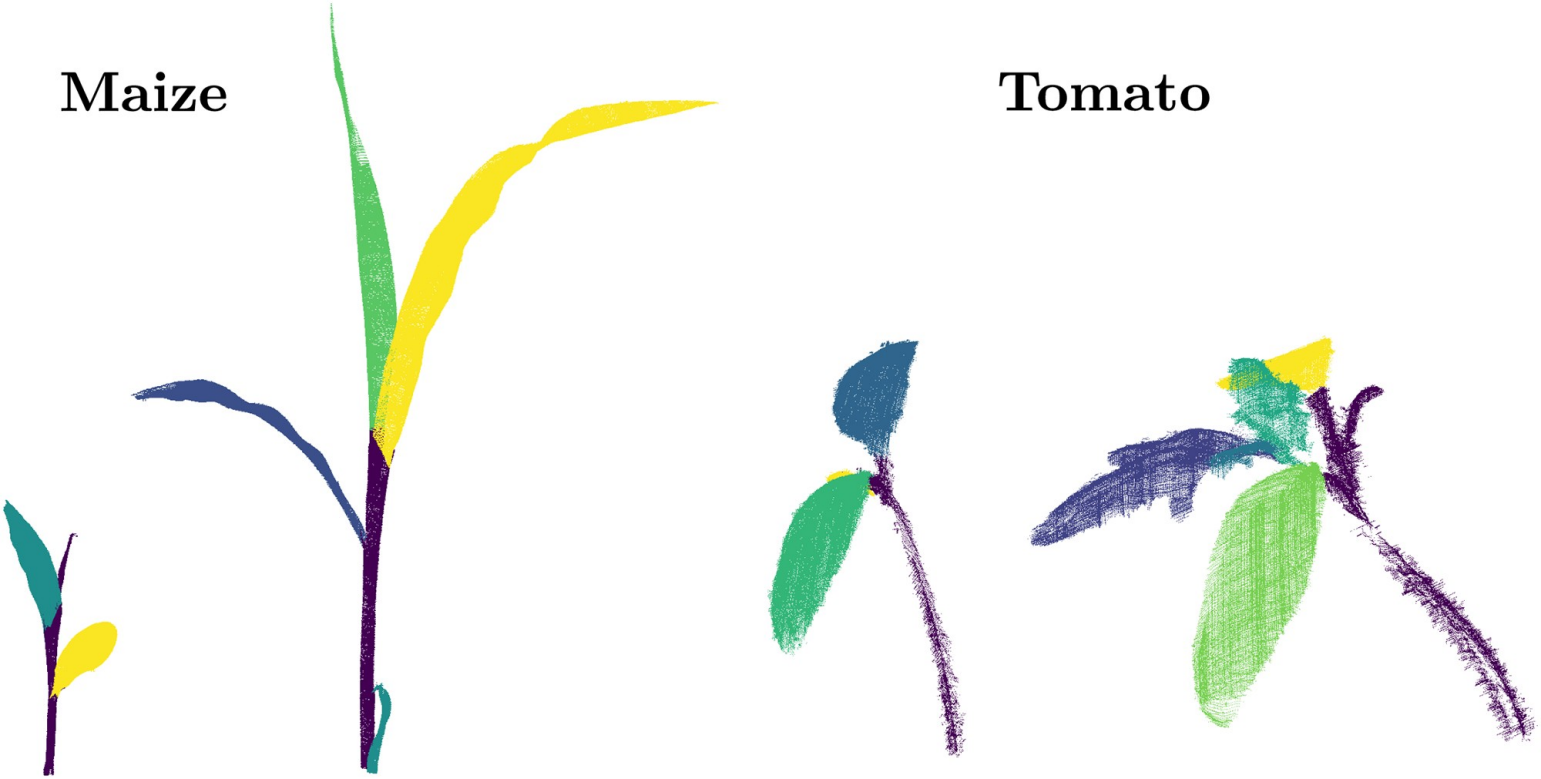
Example ground truth labels used in the evaluation of semantic classification of maize (left) and tomato (right) point clouds. The instance wise labels for each plant organ have been manually annotated by a human user.

Despite these accurate results, it is worth noticing that the ability of the SVM to classify stem points is lower on the maize dataset than on the tomato dataset. This is due to a smoother transition between stem and leaves in the maize plants. In contrast, the performance of the clustering is higher on the maize dataset. This behavior can be explained by looking at how leaves develop in the two species. While for the maize plants there is a clear separation between individual leaves, this separation is not as clear for the leaves in the tomato plants. For reference, we have shown the ground truth labels for two example plants used in the evaluation of semantic classification in Fig 6.

### 4D registration of plant point clouds

**Fig 7:**
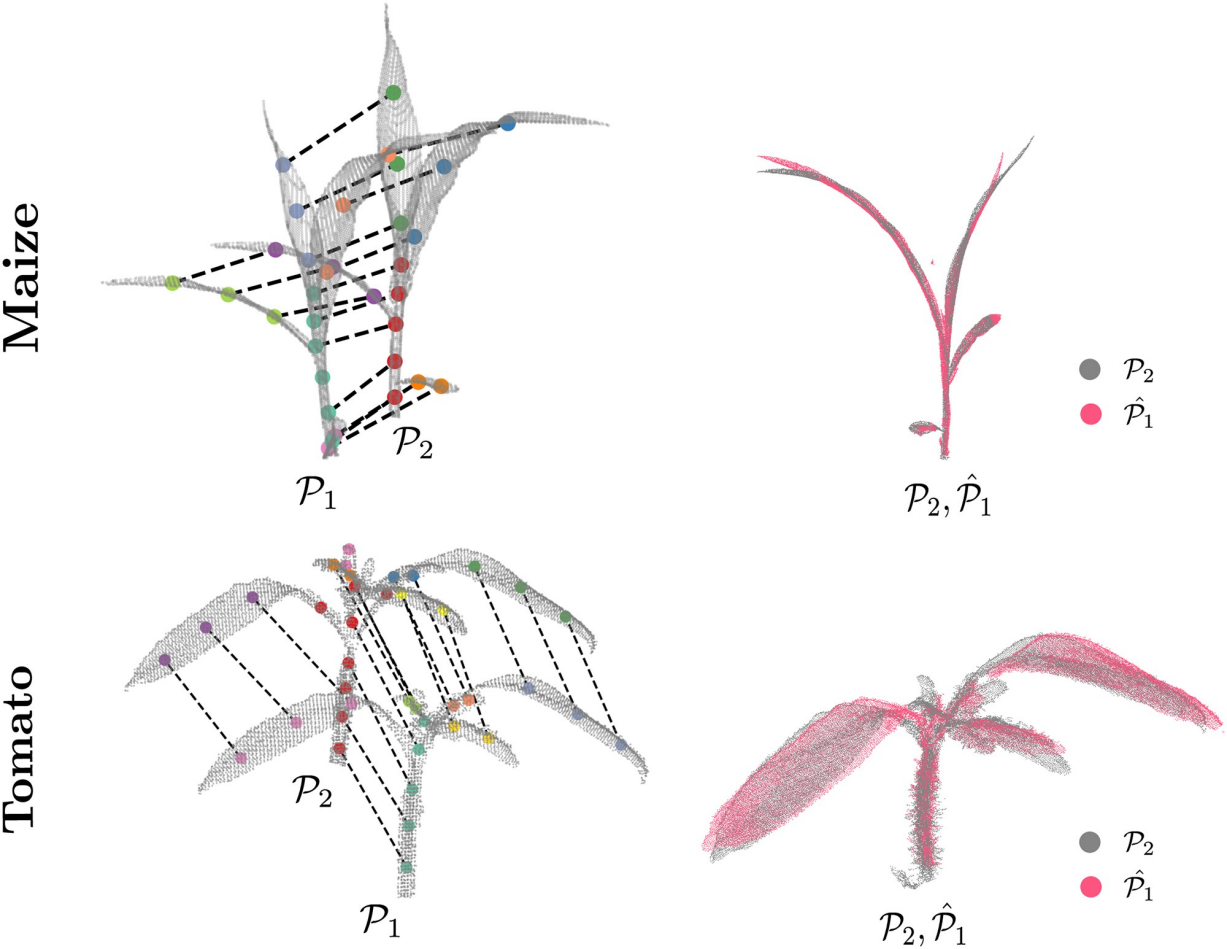
4D registration of a point cloud pair scanned on consecutive days for maize (top) and tomato (bottom) plant. The left column shows the two input point clouds ($P_{1},P_{2}$) along with their skeletons, with the estimated correspondences between the skeleton nodes shown by dashed lines, and the right column shows the deformed point cloud ${\hat{P}}_{1}$ (in pink) overlaid on $P_{2}$.

In the second experiment, we demonstrate that our approach successfully registers time-series point cloud data for two plant types. We use the same weights for our registration procedure as described in the methods section for all scan pairs (both tomato and maize datasets) at different growth stages. We also perform a quantitative evaluation of the accuracy of the registration pipeline. Fig 7 illustrates the results of the registration procedure for two example scan pairs from the maize (top) and the tomato (bottom) datasets. For both examples, we show the input point clouds ($P_{1},P_{2}$) along with their corresponding skeletons. The correspondences estimated during the registration procedure are depicted by the dashed lines joining nodes of the skeleton pair. Our approach was able to find the correspondences reliably despite the growth and the change in topology. We visualize the final registered point cloud ${\hat{P}}_{1}$ (in pink) by deforming the point cloud $P_{1}$ using the deformation parameters estimated by our approach and overlay it on the target point cloud $P_{2}$ (in gray) and observe that it overlaps well indicating that the registration results are reasonable. Most of the registered point ${\hat{P}}_{1}$ (in pink) overlaps the target point cloud $P_{2}$, however, we notice that the errors are usually high towards the outer sections of the leaves which are farther away from the skeleton curve. This effect is to be expected as the skeleton curves do not capture this area well.

Further, we quantitatively evaluate the accuracy of our registration pipeline by registering all consecutive scans of the two datasets. First, we compute the accuracy of our skeleton matching procedure by computing the percentage of correspondences estimated correctly. We define the correct correspondences as those which belong to the same organ (i.e., the same leaf or the stem) in the skeleton pair as there is no unique way to define a correct correspondence due to the growth in the plant. We manually label the different organs of the plant with a unique identifier to provide the ground truth to compute this metric. For our tomato datasets, we obtain an average of 95% correct correspondences between consecutive skeleton pairs with most pairs having all the correspondences estimated correctly. For the maize dataset, we obtain 100%of the correspondences between consecutive days correctly. Similarly, we also evaluated the accuracy of the correspondence estimation between skeleton pairs with 2 and 3 days apart from each other. For the tomato dataset, we obtain again an average of 95% correspondences with scans taken 2 days apart, whereas this falls down to 88% with scans taken 3 days apart. Again for the maize dataset, we obtain all the correspondences correctly both with skeletons taken 2 and 3 days apart. The higher accuracy for the maize plants is likely due to the simpler shape of the plant as compared to the tomato plants.

Secondly, we evaluate the accuracy of the estimated registration parameters by computing the error between the deformed source point cloud ${\hat{P}}_{1}$ and the target point cloud $P_{2}$. We define this registration error *e*_*reg*_ as:

$$\begin{array}{ccc} e_{reg} & = & \frac{1}{|{\hat{P}}_{1}|}\sum_{\begin{array}{c} i=1\\ j\in N(i) \end{array}}^{|\hat{P_{1}}|}∥{\hat{p}}_{1}^{i}-p_{2}^{j}∥, \end{array}$$ (14)

where $p_{2}^{j}$ is the nearest point to $p_{1}^{i}$ and $|{\hat{P}}_{1}|$ is the number of points in ${\hat{P}}_{1}$. For our dataset, we obtain a mean error of 3 mm with a standard deviation of 2.3 mm and a maximum error of 13 mm for consecutive scans, which indicates that the registration results are accurate.

As this registration measure may be susceptible to some degenerate cases, an extreme case being all points $p_{i}\in P_{1}$ deforming to one point in $p_{j}\in P_{2}$ giving a zero registration error. Therefore, we use the ground truth instance information to determine if such degenerated situations occur. We compute the percentage of points in *pc*_1_ that are deformed to the corresponding ground truth instances in *pc*_2_. This gives a measure of how well the instances are mapped after applying the deformation parameters. We obtain an average accuracy of 97% for the maize dataset and 89% for the tomato dataset with most of the errors occurring at the joints where leaves merge with the stem. This results suggest that the registration parameters deform the instances properly and do not result in degenerate estimates.

As a baseline comparison, we assume a rigid transformation for the whole point cloud between consecutive scans and computed the transformation with a standard ICP approach. The average error *e*_*reg*_ for this experiment is 35 mm and the maximum error is 166 mm. The large errors using a rigid transformation assumption are both due to the plant growth and in some cases, the ICP procedure diverging completely. This indicates that a rigid transformation assumption is inadequate and a non-rigid registration procedure is required to capture the growth and movement of the plant.

**Fig 8:**
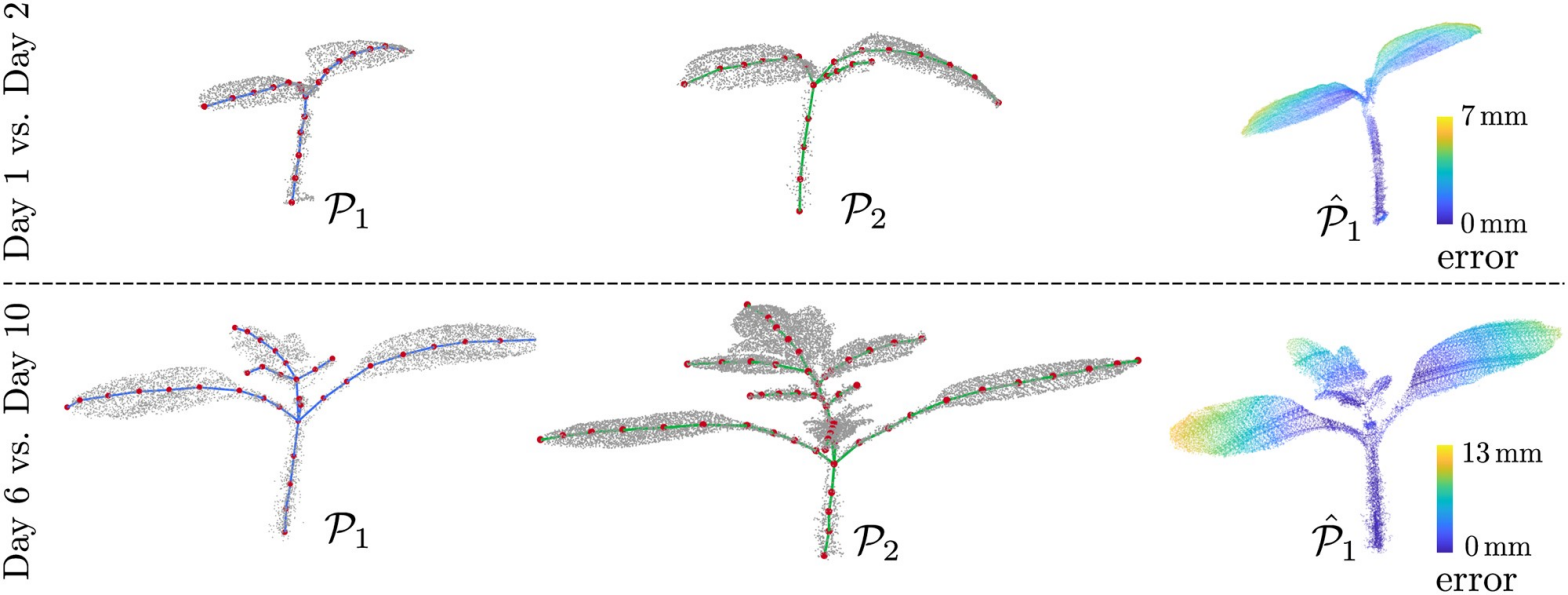
Visualizing registration error. We visualize the registration error as a heatmap for two pairs of tomato plant scans, Day 1 vs. Day 2 and Day 6 vs. Day 10. Blue represents low registration error whereas yellow represents a larger error.

We visualize the registration error as a heat map for the two example point cloud pairs in Fig 8. The heat map is projected on ${\hat{P}}_{1}$ to show how well different portions of the plant are registered. The blue regions in the heat map represent a smaller registration error whereas the yellow regions indicate large errors. Most of the regions are blue indicating a successful registration, however, we notice that the errors are usually high towards the outer sections of the leaves which are farther away from the skeleton curve. This effect is to be expected as the skeleton curves do not capture this area well.

### Temporal tracking of phenotypic traits

**Fig 9:**
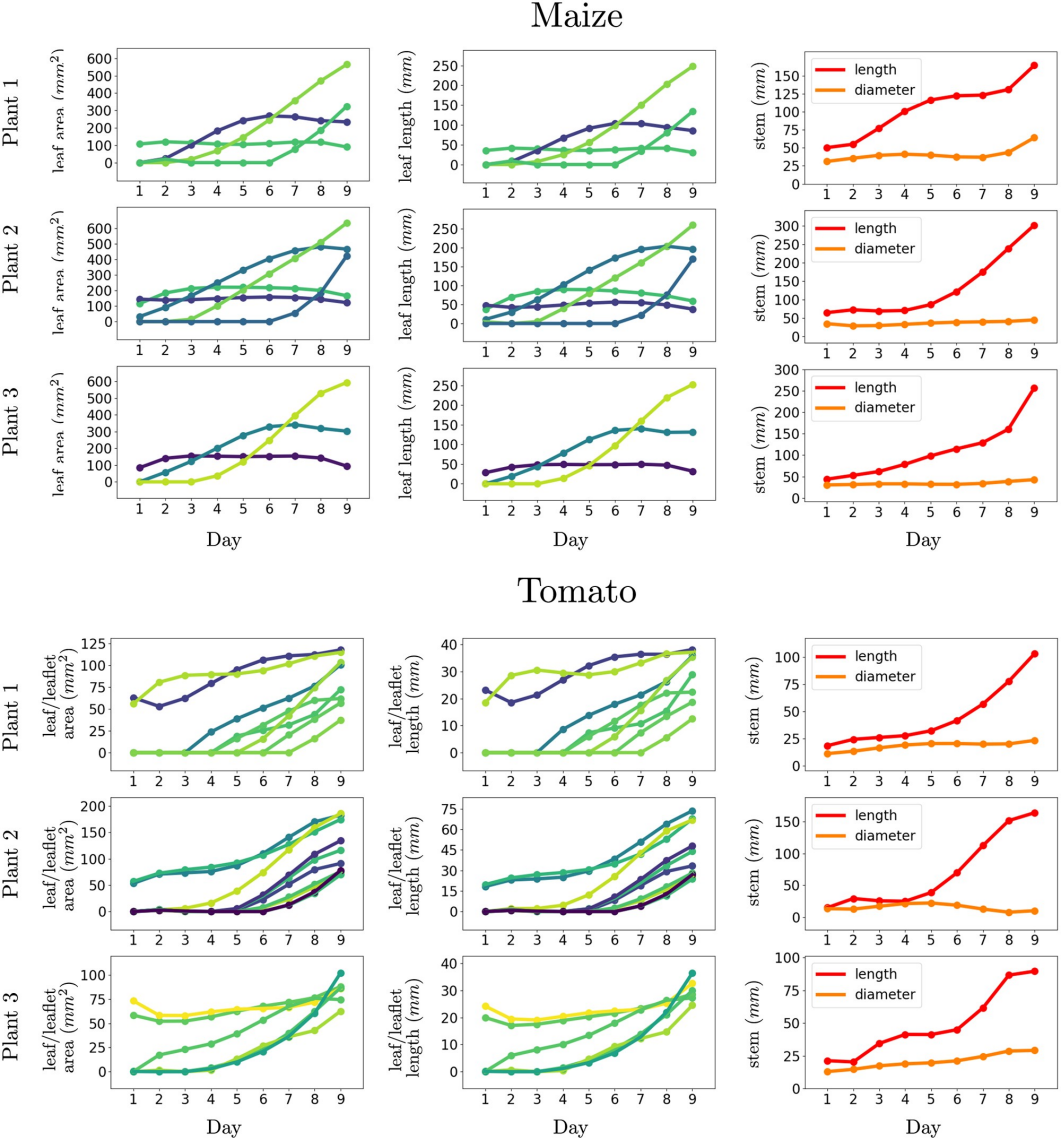
Tracking phenotypic traits for individual organs of the plant. Our registration procedures allows us to track the growth of the stem and different leave lengths over time and detect topological events such as the emergence of new leaves. Different shades of blue and green in these plots represent individual leaf instances in the first two columns. The orange and red represent the length and diameter of the stem respectively.

In this experiment, we show that the spatial-temporal registration results computed by our approach allows us to compute several phenotypic traits and track them temporally. We compute the area *l*_*a*_ and length *l*_*l*_ for leave instances and the diameter *s*_*d*_ and length *s*_*l*_ of the stem for each point cloud in the temporal sequence as describe previously, and associate it over time using the data associations estimated by our approach during the registration process. The tracking results for the three sample plants from the maize and tomato datasets are visualized in Fig 9. The first two columns in Fig 9, track the leaf area and leaf length over time. Different shades of blue and green in these plots represent individual leaf instances. In addition, we can also detect certain events, which mark a topological change in the structure of the plant such as the appearance of a new leaf. These events can be recognized from the leaf area or leaf length plots in Fig 9 whenever a new line rises up from the zero level. For example, for the first plant in the maize dataset, a new leaf emerges on day 2, 3, and 6. In the rightmost column in Fig 9, we see that stem length and diameter for both the datasets increase considerably over the data acquisition period. Such phenotypic information can also be used to compute the BBCH scale [83] of the plant, which is a growth stage scale and provides valuable information to the agronomists.

### Temporal interpolation of plant point clouds

**Fig 10:**
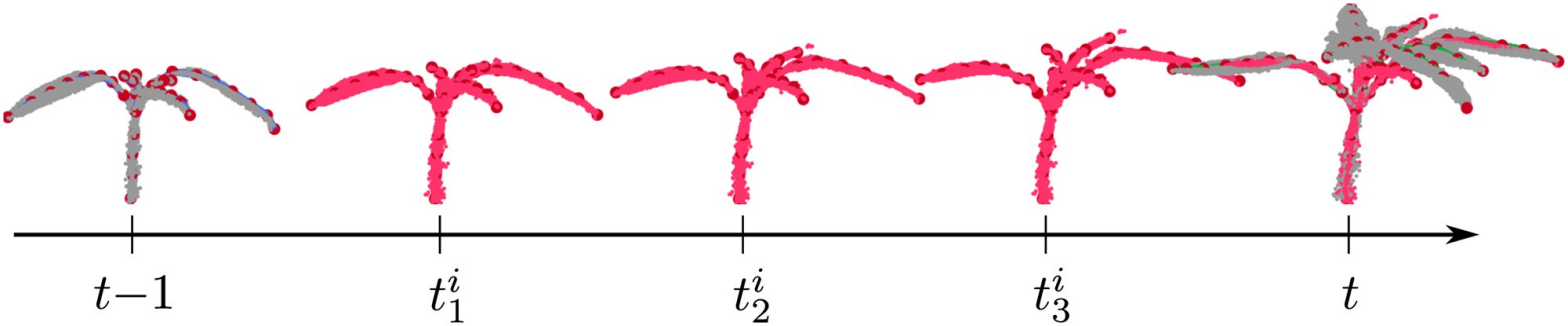
Interpolation of point clouds at intermediate time intervals. Point clouds (gray) at time *t* − 1 and *t* come from actual scan measurements whereas the points clouds (pink) at time instants $t_{1}^{i},t_{2}^{i},t_{3}^{i}$ visualize the three interpolated scans.

In the last experiment, we demonstrate that the registration results can be used to interpolate the point clouds at intermediate points in time, i.e., in between the actual acquisition times of the scans. The ability to interpolate is useful for analyzing properties of the plants even when actual measurements are not available. It allows us to predict both, the motion and growth at intermediate time intervals. We visualize the interpolated point cloud at three time instances $t_{1}^{i},t_{2}^{i},t_{3}^{i}$ between the two scans in top of Fig 10. This allows us to animate a time-lapse view of the plants. The pink point clouds represent the interpolated scans and overlaps well with point cloud (gray) at time *t* indicating that the interpolation is reasonable. As the interpolation procedure does not actually model the movement or the growth of the plant, the result of the interpolation may differ from the actual plant at those instances. In order to evaluate the interpolation step, we take the scans on day *t* − 1 and day *t* + 1, then interpolate the point cloud at day *t* and compare against the actual point cloud on day *t*. We compute the registration error (as described in (14)) and obtain a mean *e*_*reg*_ of 4 mm with a standard deviation of 1.9 mm. We also compute the percentage of points in $P_{t-1}$ that are registered to the corresponding ground truth instances in $P_{t}$ similar to the measure in the registration experiments. We obtain an average accuracy of 91% for the maize dataset and 83% suggesting that our interpolation is a reasonable approximation of the real plant growth.

## Summary and conclusion

In this paper, we presented a novel approach for spatio-temporal registration of 3D point clouds of individual plants. The proposed method works for raw sensor data stemming from a range sensor such as a 3D LiDAR or a depth camera. The processing of the sensor data happens in a fully automated fashion without manual intervention. We implemented and evaluated our approach on datasets of tomato and maize plants presenting challenging situations and supported all claims made in this paper. The experiments suggest that our registration approach can be used as a basis for tracking plant traits temporally and contribute towards automated phenotyping. Furthermore, we released the code of our approach as open-source software.

Our approach works as follows: First, it estimates the skeletal structure of the plant, exploiting also a point-wise classification approach to compute a skeleton representing the plant. This skeleton structure including the semantic information is used to find reliable correspondences between parts of the plant recorded at different points in time using a novel data association approach that relies on a hidden Markov model. It allows for estimating a global association between the skeletons of the plants extracted from point clouds recorded at different points in time. As our results showcase, this approach can deal with changing appearance and changing topology of the 3D structure of the plant. This is an essential capability to form a robust alignment of the 4D data, i.e., of 3D geometry and time. Given the data associations, we explicitly model the deformation and growth of the plant over time using multiple affine transformations associated with the individual nodes of the skeleton structure. In this way, individual parts of the plant are transformed using different affine transformation modeling the different growth along the plant. The parameters for these transformations are estimated using a robust least-squares approach including regularizations. Given the resulting parameters, we can align 3D scans taken at different points in time and transform them according to the growth. This, in turn, allows us to estimate basic phenotypical parameters such as leaf area, leaf length, as well as stem diameter or length and track their development over time in an automated fashion.

## Supporting information

S1 Video Interpolation of plant point clouds. The video shows the interpolation of the plant point clouds for time instances in between actual measurements.
(MP4)

S1 Fig (PDF)

S2 Fig (PDF)

S3 Fig (PDF)

S4 Fig (PDF)
