# Supplementary figures and images for: Registration of spatio-temporal point clouds of plants for phenotyping
Source: PLoS One. 2021 Feb 25;16(2):e0247243. doi: 10.1371/journal.pone.0247243 (PMC7906482; doi:10.1371/journal.pone.0247243)

# Maize

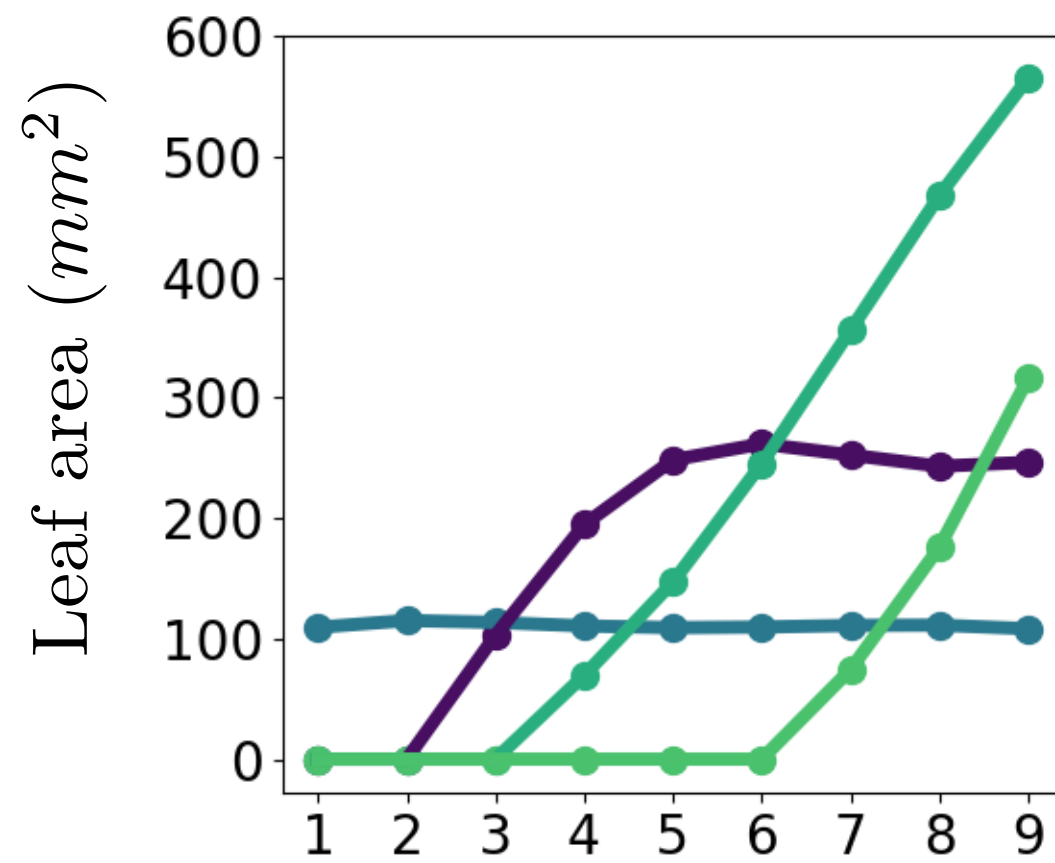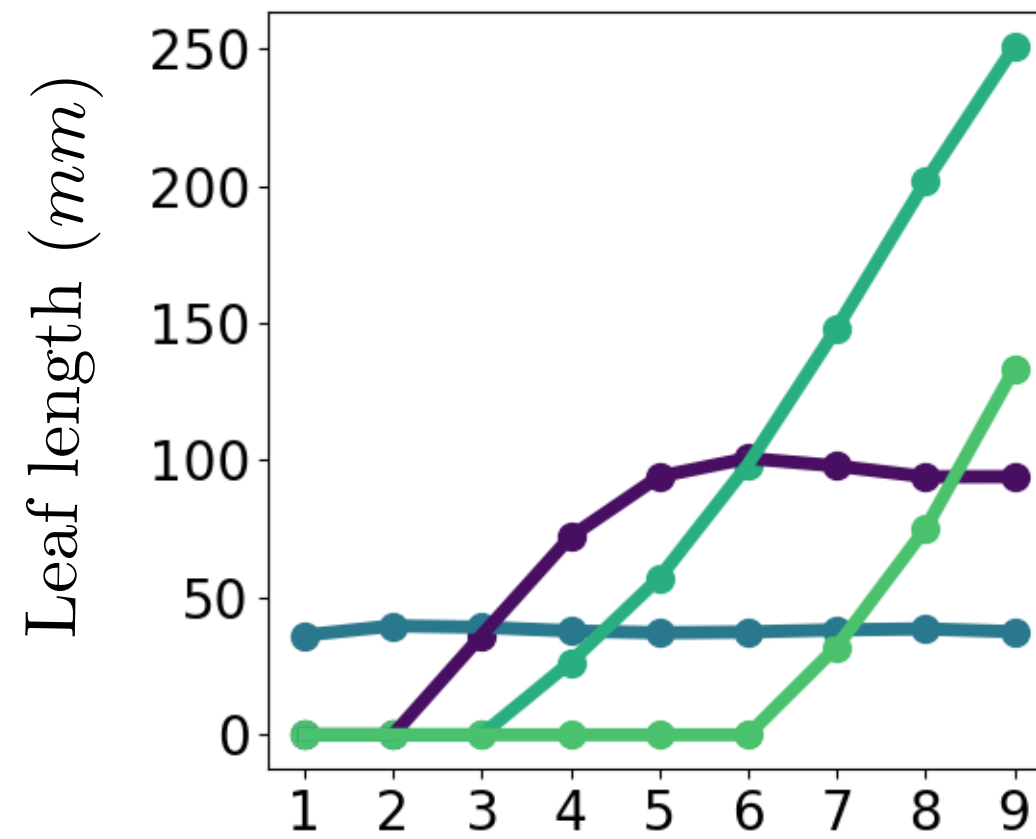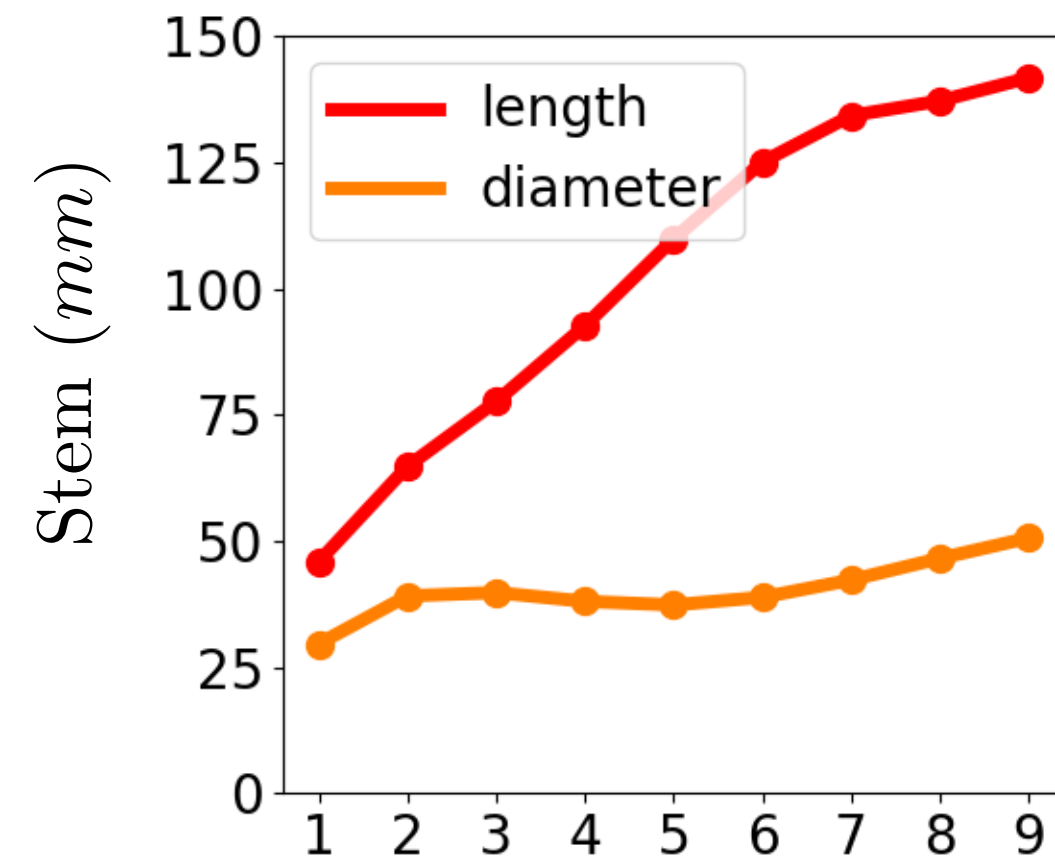

# Tomato

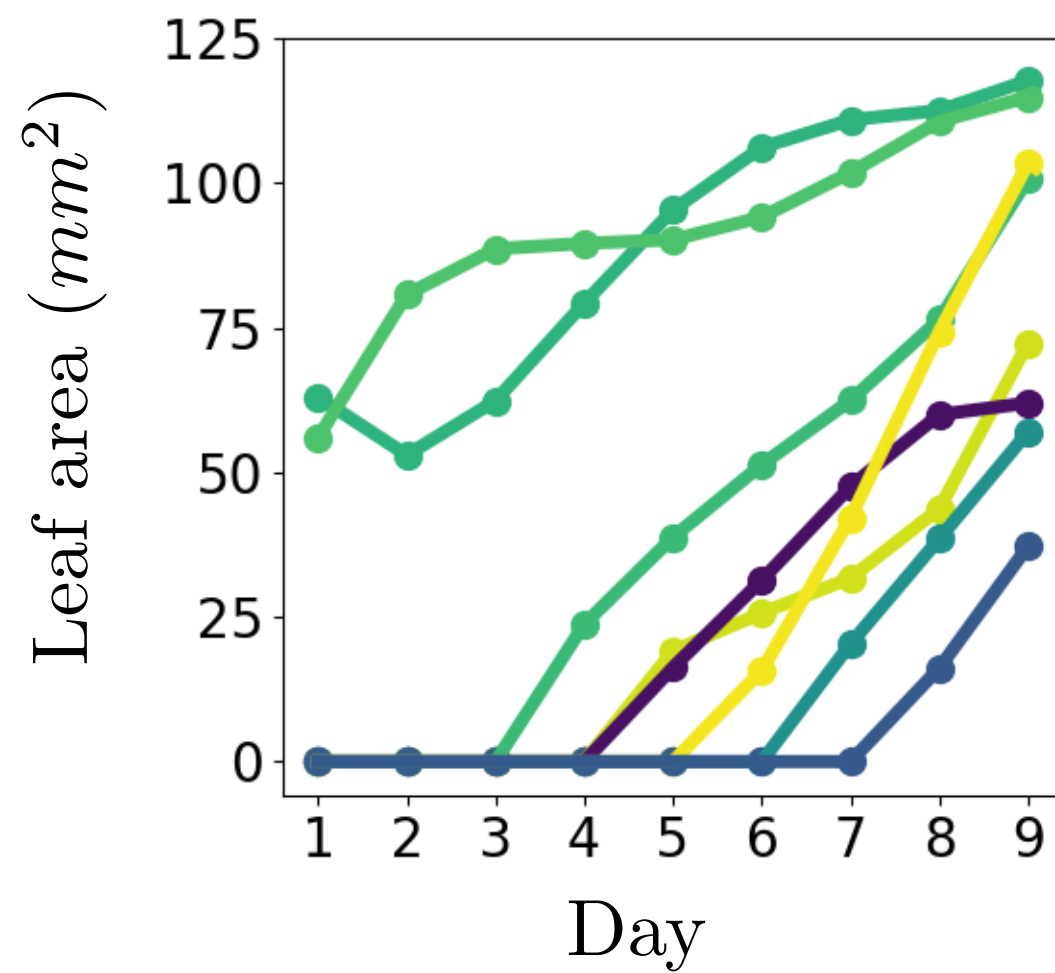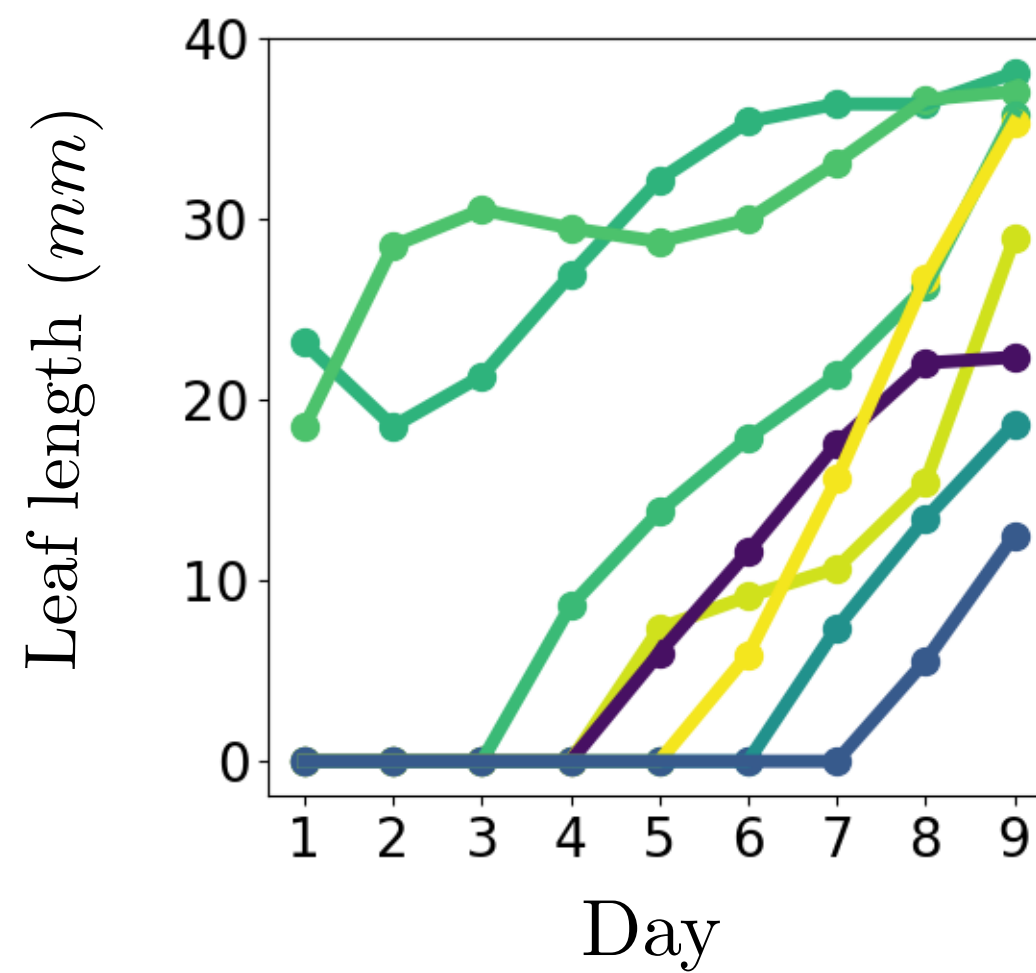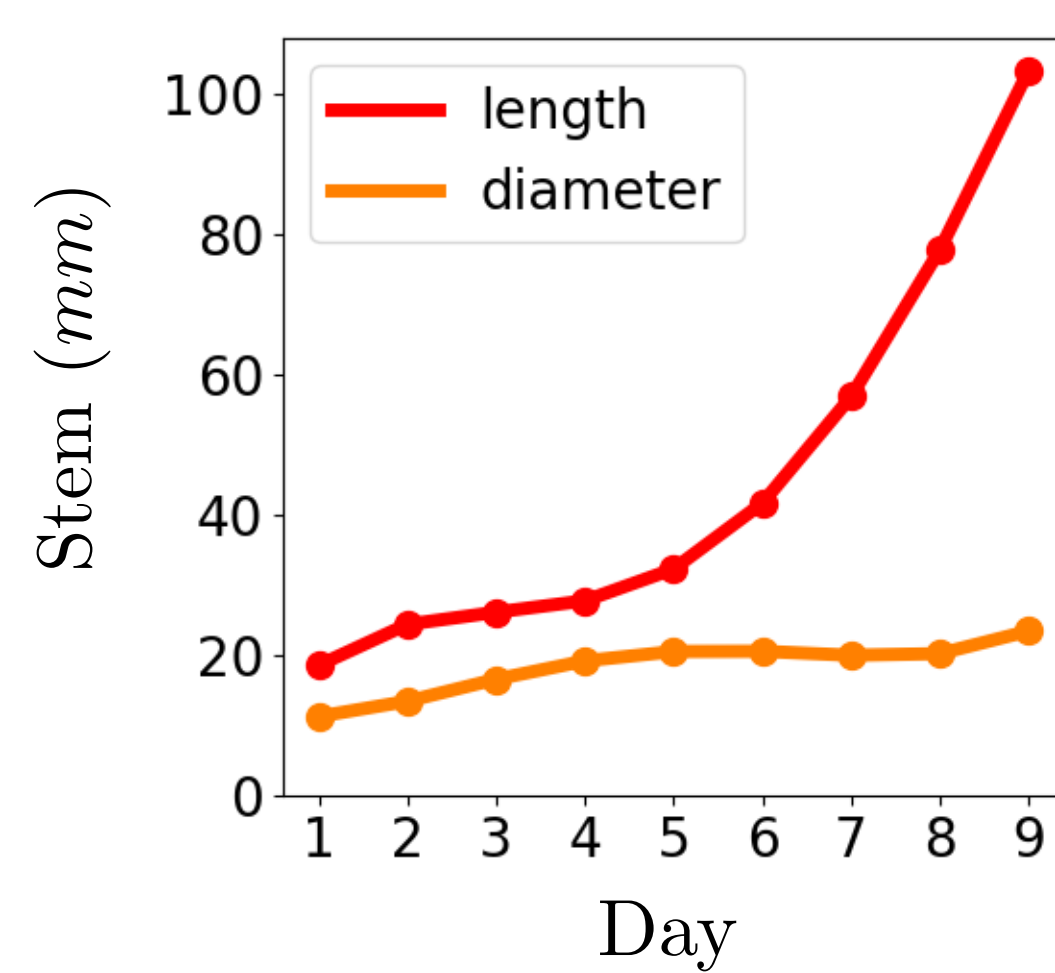

Supplement: S1 Fig — (PDF) [file pone.0247243.s002.pdf]

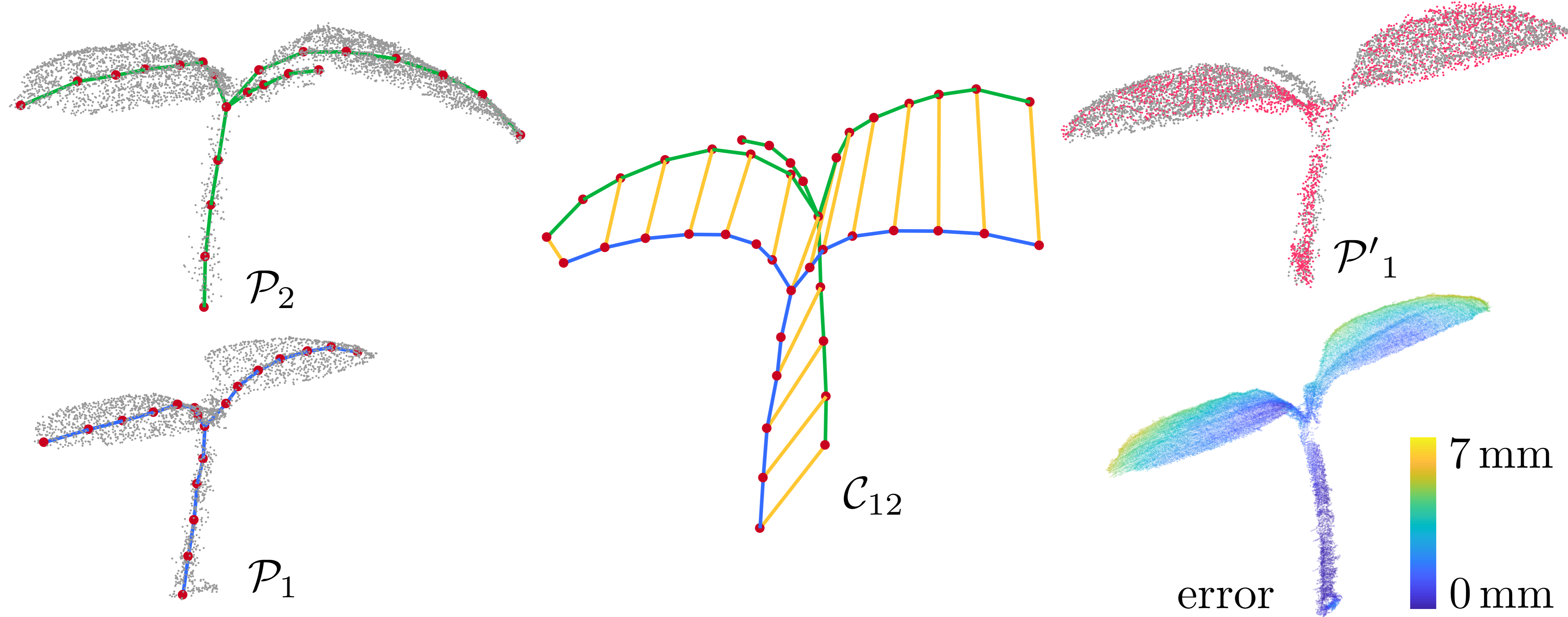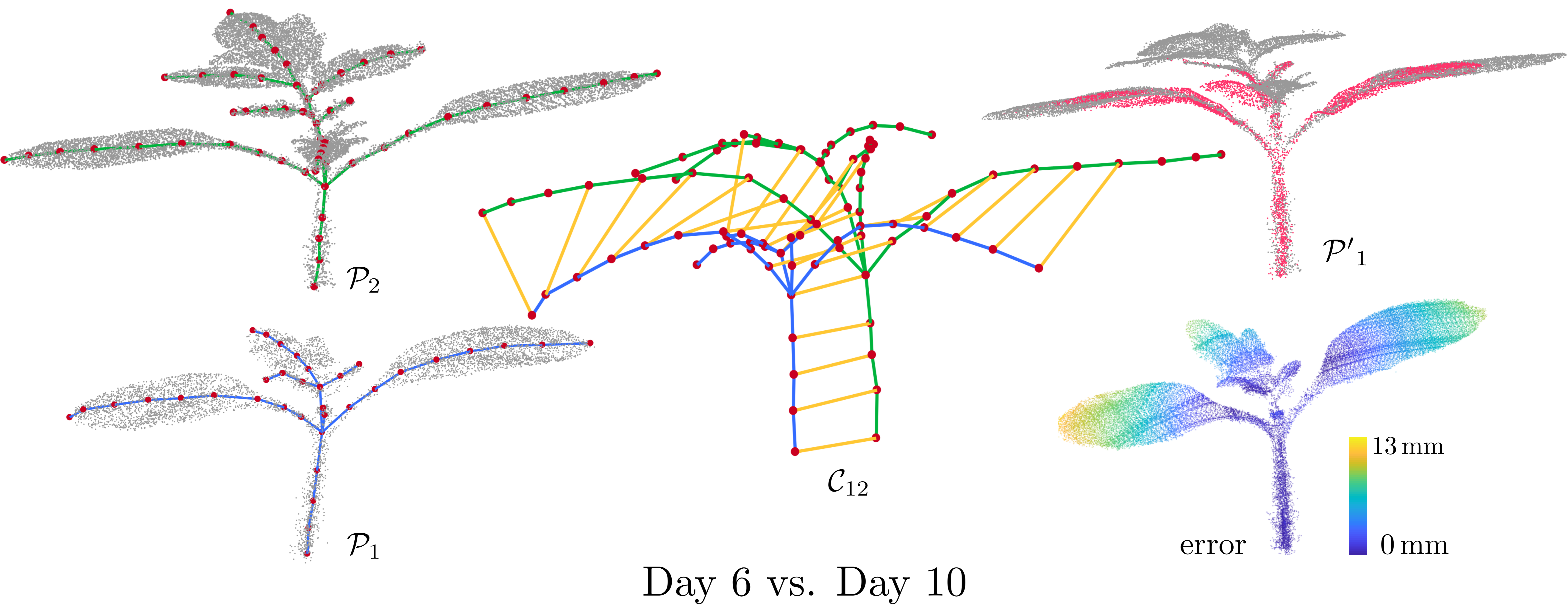

Supplement: S2 Fig — (PDF) [file pone.0247243.s003.pdf]

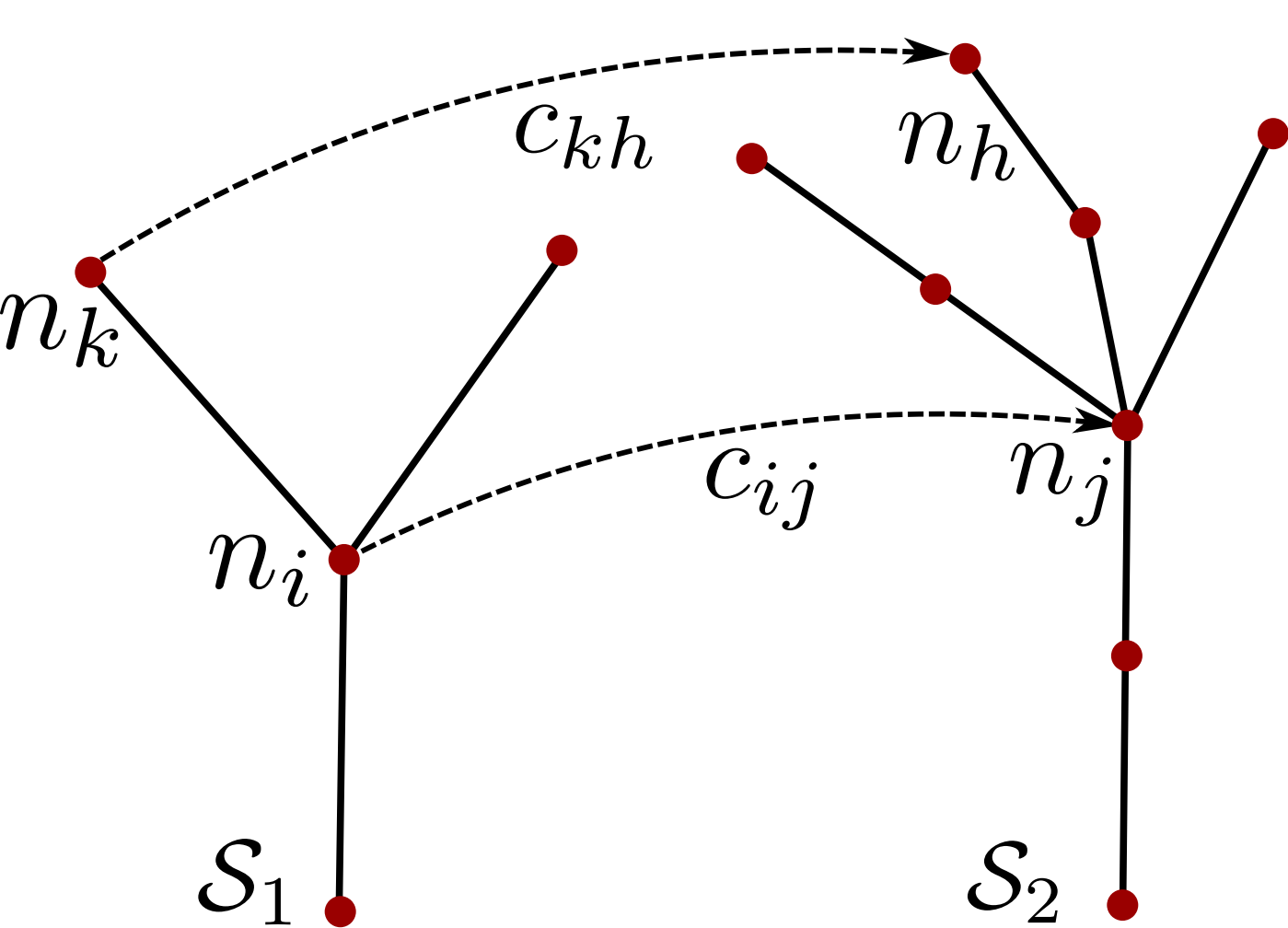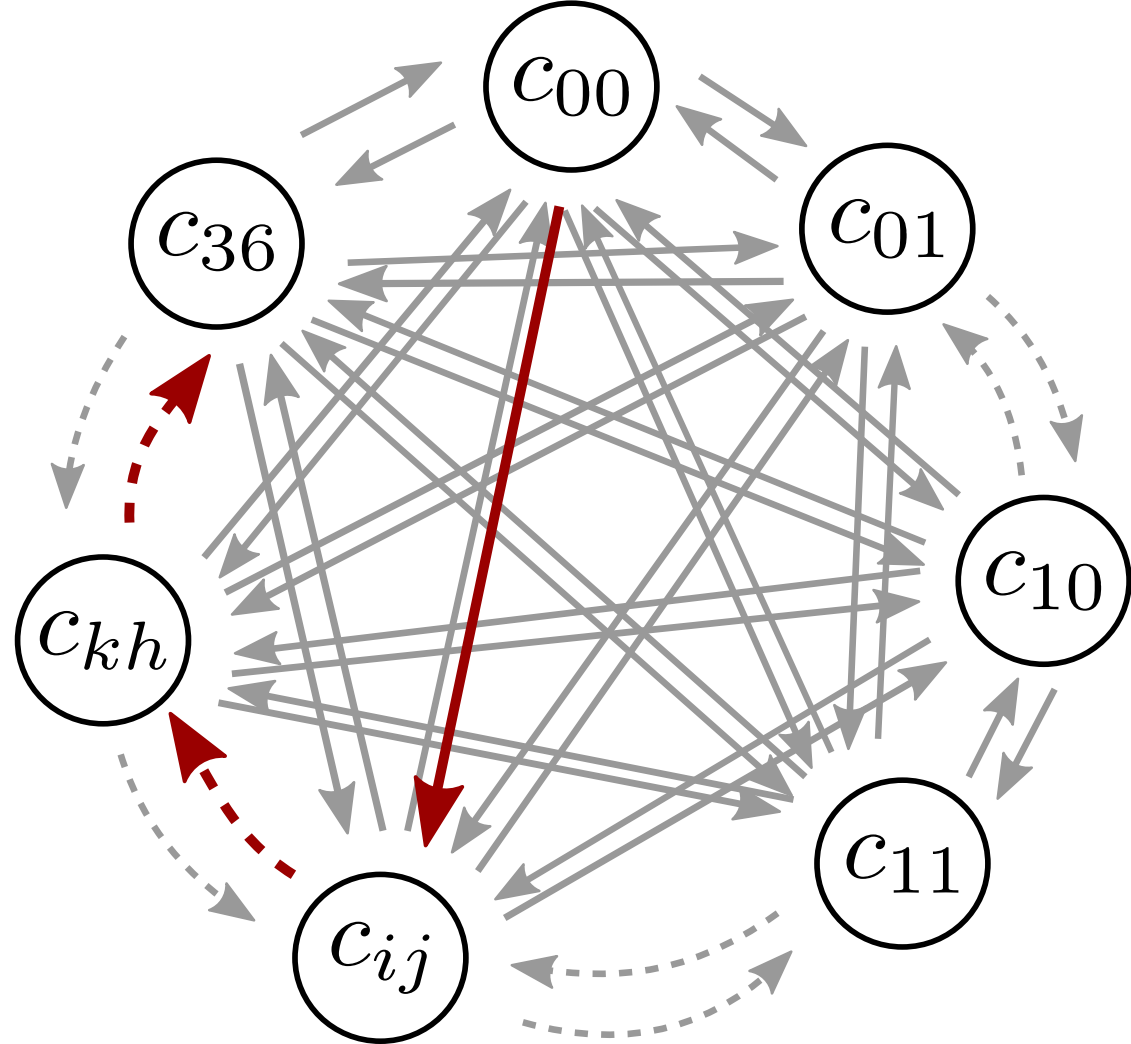

Supplement: S3 Fig — (PDF) [file pone.0247243.s004.pdf]

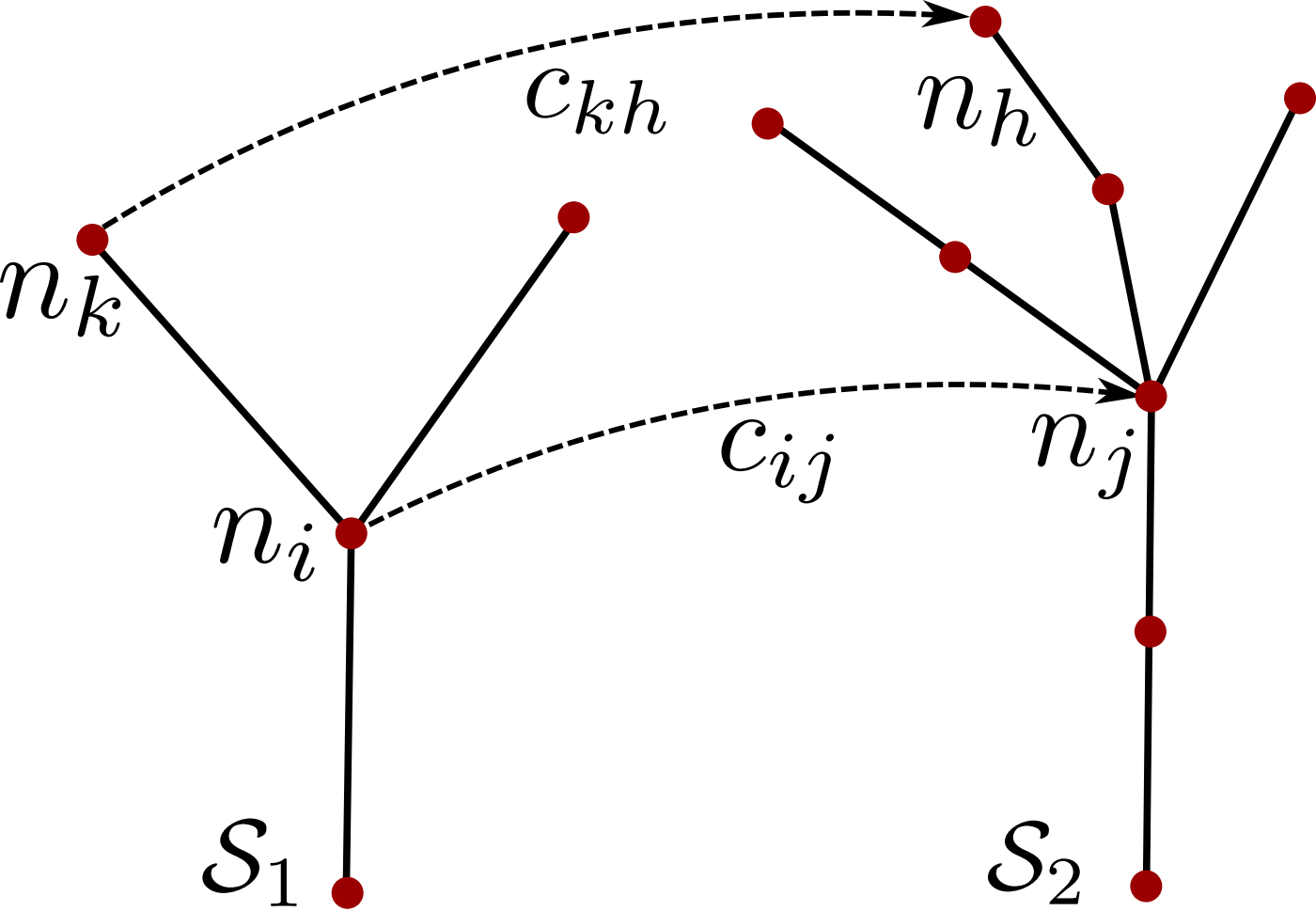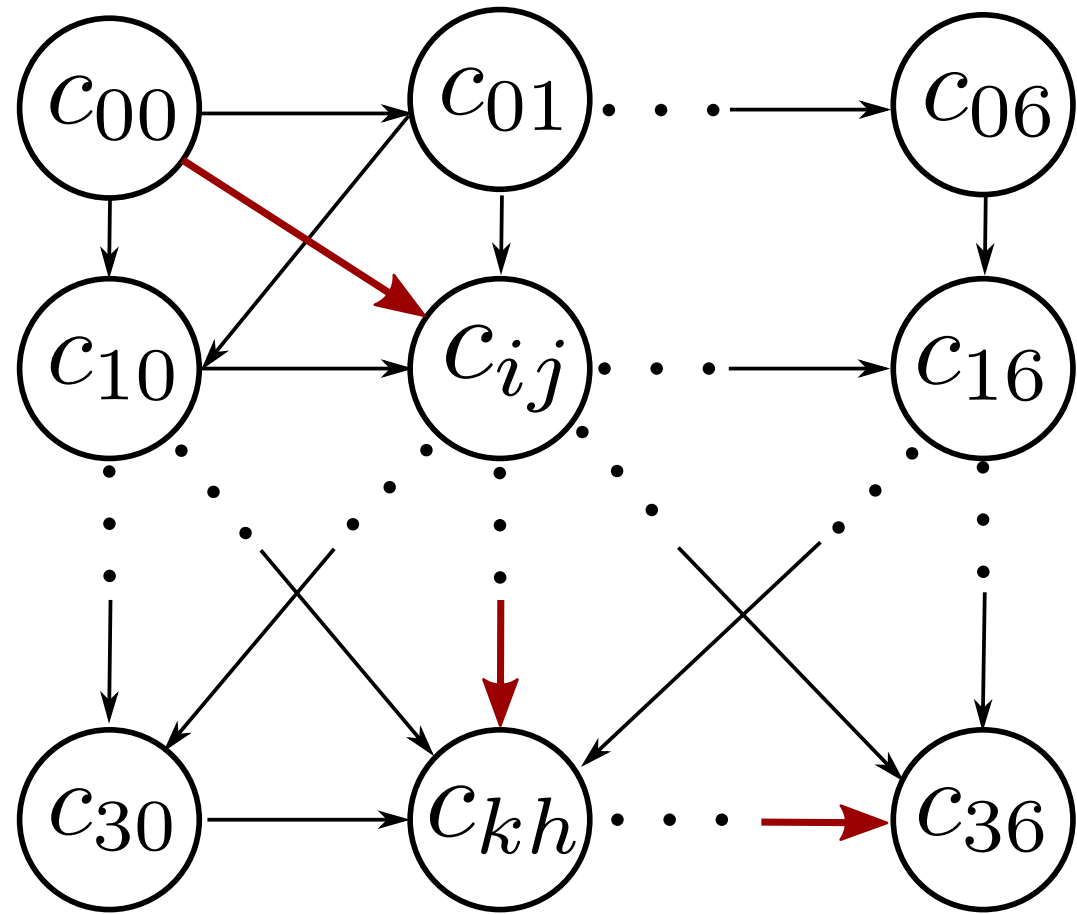

Supplement: S4 Fig — (PDF) [file pone.0247243.s005.pdf]
